# Influences of Alkyl and Aryl Substituents on Iminopyridine Fe(II)- and Co(II)-Catalyzed Isoprene Polymerization

**DOI:** 10.3390/polym8110389

**Published:** 2016-11-03

**Authors:** Lihua Guo, Xinyu Jing, Shuoyan Xiong, Wenjing Liu, Yanlan Liu, Zhe Liu, Changle Chen

**Affiliations:** 1 School of Chemistry and Chemical Engineering, Qufu Normal University, Qufu 273165, China; 18753770200@163.com (L.G.); m18463758798@163.com (X.J.); liuwenjingaaa@126.com (W.L.); lyl373740@163.com (Y.L.); liuzheqd@163.com (Z.L.); 2 Key Laboratory of Soft Matter Chemistry, Chinese Academy of Sciences, Department of Polymer Science and Engineering, University of Science and Technology of China, Hefei 230026, China; xiong18@mail.ustc.edu.cn

**Keywords:** iminopyridine, Iron(II), Cobalt(II), isoprene polymerization, selectivity

## Abstract

A series of alkyl- and aryl-substituted iminopyridine Fe(II) complexes **1a**–**7a** and Co(II) complexes **2b**, **3b**, **5b**, and **6b** were synthesized. The activator effect, influence of temperature, and, particularly, the alkyl and aryl substituents’ effect on catalytic activity, polymer molecular weight, and regio-/stereoselectivity were investigated when these complexes were applied in isoprene polymerization. All of the Fe(II) complexes afforded polyisoprene with high molecular weight and moderate *cis*-1,4 selectivity. In contrast, the Co(II) complexes produced polymers with low molecular weight and relatively high *cis*-1,4 selectivity. In the iminopyridine Fe(II) system, the alkyl and aryl substituents’ effect exhibits significant variation on the isoprene polymerization. In the iminopyridine Co(II) system, there is little influence observed on isoprene polymerization by alkyl and aryl substituents.

## 1. Introduction

The polymerization of isoprene can afford polymers with various regio- and/or stereoregularities such as isotactic or syndiotactic polyisoprene via 1,2 or 3,4 addition, and *cis*- or *trans*-1,4 polyisoprene via 1,4 addition. The structures of polyisoprene strongly influence the properties of the resulting material. For example, the properties of *cis*-1,4 polyisoprene is very similar to those of natural rubber [1], while the properties of *trans*-1,4 polyisoprene is very close to those of gutta-percha [2]. The development of highly efficient and highly regio- and stereoselective catalysts plays a key role in the field of metal-catalyzed polymerization of conjugate dienes [3]. Titanium and rare-earth metal catalysts can afford *cis*-1,4 and *trans*-1,4 polybutadienes and polyisoprenes with up to 98% selectivity [4,5,6,7,8,9,10,11,12,13,14,15]. In addition, some late transition-metal catalytic systems were successfully applied in olefins [16,17,18,19,20,21,22,23,24,25,26,27,28,29,30,31,32,33,34], butadiene [4,35,36,37,38,39,40,41,42,43,44,45], and isoprene [36,37,46,47,48,49,50] polymerization. Late transition-metal catalysts have lower Lewis acid characteristics and may possess high tolerance towards functional groups and polar additives. Special attention was paid to low-cost and earth-abundant iron- and cobalt-based catalysts with well-defined molecular structures that could be easily prepared.

Recently, Dai et al. [41] showed that an aryl-substituted iminopyridine Co(II) catalyst exhibited high catalytic activity and *cis*-1,4-selectivity for 1,3-butadiene polymerization. Raynaud et al. [51] reported that the combination of the iminopyridine Fe(II) complexes, alkylaluminum, and dealkylating reagent [Ph_3_C][B(C_6_F_5_)_4_] can polymerize isoprene with high stereoselectivity. The octyl-substituted imines favor *trans*-1,4 insertion, whereas supermesityl-substituted imines favor *cis*-1,4 insertion. The authors suggested that higher electron density at the iron center may increase the *trans*-1,4 selectivity. However, this accidental discovery and studies of only these two catalysts make it difficult draw any rational conclusions.

**Scheme 1:**
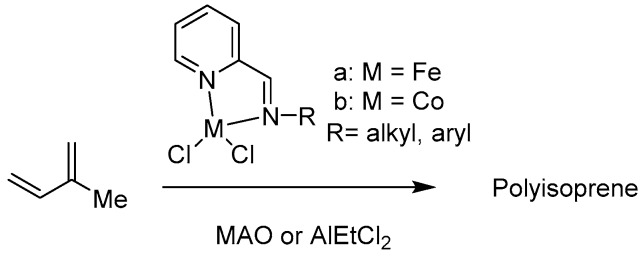
Alkyl- and aryl-substituted iminopyridine Fe(II) and Co(II) complexes for isoprene polymerization.

Inspired by these works, we became very interested in the influence of iminopyridine ligand substituents on the selectivity of isoprene polymerization. In this work, various alkyl- and aryl-substituted iminopyridine Fe(II) and Co(II) complexes were synthesized and employed in isoprene polymerization when activated using an alkylaluminum (methylaluminoxane (MAO) or AlEtCl_2_) (Scheme 1). The effects of the imine moiety on the catalytic activity, molecular weight, and, particularly, the regio- and stereoselectivity were investigated.

## 2. Experimental Section

### 2.1. General Information

All manipulations of air-and-moisture sensitive materials were performed under a dry nitrogen atmosphere by using standard Schlenk techniques. Nitrogen was purified by passing through a MnO oxygen-removal column and an activated 4 Å molecular sieve column. ^1^H and ^13^C NMR spectra were recorded using CDCl_3_ as solvent on a Bruker Ascend™ 500 spectrometer (Bruker, Karlsruhe, Germany) at room temperature unless otherwise stated. The chemical shifts of the ^1^H and ^13^C NMR spectra (Bruker, Karlsruhe, Germany) were referenced to tetramethylsilane (TMS). Coupling constants are in units of hertz. Fourier-transform infrared (FTIR) spectrometry was performed on a Thermo Scientific Nicolet iS5 (Thermo Fisher Scientific Corporation, Waltham, MA, USA) using the conventional KBr wafer technique. Elemental analysis was performed by the Analytical Center of the University of Science and Technology of China (Hefei, China). Mass spectra were recorded on a P-SIMS-Gly of Bruker Daltonics Inc. (EI, Bruker Daltonics Inc., Billerica, MA, USA). X-ray Diffraction data were collected at 298(2) K on a Bruker Smart CCD area detector (Bruker, Karlsruhe, Germany) with graphite-monochromated MoKα radiation (λ = 0.71073 Å). Molecular weights and molecular weight distributions were determined by gel permeation chromatography (GPC, Waters, Milford, MA, USA) employing a series of two linear Styragel columns (HR2 and HR4) at an oven temperature of 45 °C. A Waters 1515 pump and Waters 2414 differential refractive index detector (30 °C) were used. The eluent was tetrahydrofuran (THF) at a flow rate of 1.0 mL·min^−1^. A series of low-polydispersity polystyrene standards was used for calibration. Hexane (Tianjin Fuyu Fine Chemical Limited Company, Tianjin, China), toluene (Laiyang Fine Chemical Factory, Laiyang, China) and THF (tetrahydrofuran, Tianjin Fuyu Fine Chemical Limited Company, Tianjin, China) were refluxed over sodium benzophenone ketyl until the solution turned blue and then distilled before use. CH_2_Cl_2_ (Tianjin Fuyu Fine Chemical Limited Company, Tianjin, China) was refluxed over phosphorus pentoxide for 8 h and distilled under a nitrogen atmosphere. Isoprene (Aladdin Industrial Corporation, Shanghai, China) was dried over CaH_2_ prior to use in polymerization. Ligands **L2** and **L5**–**L7** were prepared according to reported procedure [51,52,53]. Complexes **2a**, **5a**, **6a**, and **6b** were synthesized according the reported method [41,51,54]. All other reagents were purchased from commercial sources and used without purification.

### 2.2. General Procedure for the Synthesis of Ligands ***L1***, ***L3***, and ***L4***

A solution of the corresponding amine (30 mmol) in methanol (30 mL) was added to pyridine-2-carbaldehyde (30 mmol) and a drop of formic acid was subsequently added. The mixture was stirred at room temperature overnight.

*Cyclohexyl(pyridin-2-yl-methylene)amine* (**L1**): the reaction mixture was concentrated under reduced pressure. The residue was purified by distillation under vacuum to give the colorless oil. Yield: 5.37 g (95.1%). ^1^H NMR (500 MHz, CDCl_3_) δ 8.63 (*d*, *J* = 3.9 Hz, 1H), 8.40 (*s*, 1H, CH=N), 7.99 (*d*, *J* = 7.8 Hz, 1H), 7.70 (*dd*, *J* = 10.6, 4.1 Hz, 1H), 7.43–7.13 (*m*, 1H), 3.53–3.19 (*m*, 1H, N–CH), 2.05–1.52 (*m*, 7H), 1.53–1.04 (*m*, 3H). ^13^C NMR (126 MHz, CDCl_3_) δ 157.97 (CH=N), 153.79, 147.76, 134.52, 122.80, 119.47, 67.83, 32.84, 24.30, 23.13. Anal. calcd. for C_12_H_16_N_2_: C, 76.55; H, 8.57; N, 14.88; found: C, 76.13; H, 8.44; N, 14.79.

*Adamantyl(pyridin-2-yl-methylene)amine* (**L3**): the reaction mixture was concentrated under reduced pressure. The residue was purified by distillation under vacuum to give the light-yellow oil which quickly changed to solid at room temperature. Yield: 6.76 g (93.7%). ^1^H NMR (500 MHz, CDCl_3_) δ 8.63 (*d*, *J* = 3.6 Hz, 1H), 8.36 (*s*, 1H, CH=N), 8.01 (*t*, *J* = 16.1 Hz, 1H), 7.73 (*t*, *J* = 7.3 Hz, 1H), 7.35–7.27 (*m*, 1H), 2.23–2.13 (*m*, 3H, CH(CH_2_)_3_), 1.83 (*s*, 6H, CH(CH_2_)_3_), 1.79–1.65 (*m*, 6H, CH(CH_2_)_3_). ^13^C NMR (126 MHz, CDCl_3_) δ 156.08 (CH=N), 155.58, 149.17, 136.38, 124.24, 120.82, 58.02, 42.89, 36.44, 29.44. Anal. calcd. for C_16_H_20_N_2_: C, 79.96; H, 8.39; N, 11.66; found: C, 79.81; H, 8.37; N, 11.72.

*Triphenyl(pyridin-2-yl-methylene)amine* (**L4**): the white solid precipitated from the solution and was separated by filtration. The white solid was washed with methanol (3 × 5 mL). Yield: 8.18 g (78.3%). ^1^H NMR (500 MHz, CDCl_3_) δ 8.61 (*d*, *J* = 3.9 Hz, 1H), 8.38 (*d*, *J* = 7.9 Hz, 1H, CH=N), 7.99 (*s*, 1H), 7.81 (*t*, *J* = 7.5 Hz, 1H), 7.34 (*m*, 16H, ). ^13^C NMR (126 MHz, CDCl_3_) δ 160.95 (CH=N), 155.27, 149.28, 145.25, 136.61, 129.76, 127.85, 126.93, 124.84, 121.32. Anal. calcd. for C_25_H_20_N_2_: C, 86.17; H, 5.79; N, 8.04; found: C, 86.32; H, 5.63; N, 7.98.

### 2.3. General Procedure for the Synthesis of Iron Complexes

All complexes were prepared in a similar manner by the reaction of anhydrous FeCl_2_ with the corresponding ligands in dichloromethane. A typical synthetic procedure used for complexes **1a**, **3a**, **4a**, and **7a** is as follows. Ligand (1.0 mmol) and FeCl_2_ (1.0 mmol) were stirred in 10 mL of dichloromethane overnight at room temperature. The precipitate was collected by filtration, washed with hexane (10 mL × 2) and dried under vacuum to obtain orange, purple, or burgundy solid.

*(^Cyclohexyl^Iminopyridine)FeCl_2_* (**1a**) (purple solid, 0.30 g, 95%): MALDI-TOF-MS (*m*/*z*): calcd. for C_12_H_16_ClFeN_2_: 279.0351, found: 278.9959 [M − Cl]^+^. Anal. calcd. for C_12_H_16_Cl_2_FeN_2_: C, 45.75; H, 5.12; N, 8.89; found: C, 46.20; H, 4.99; N, 9.12. IR/cm^−1^:1563, ν(C=N).

*(^Adamantyl^Iminopyridine)FeCl_2_* (**3a**) (orange solid, 0.35 g, 95%): MALDI-TOF-MS (*m*/*z*): calcd. for C_16_H_20_ClFeN_2_: 331.0664, found: 330.9991 [M − Cl]^+^. Anal. calcd. for C_16_H_20_Cl_2_FeN_2_: C, 52.35; H, 5.49; N, 7.63; found: C, 52.55; H, 5.33; N, 7.29. IR/cm^−1^:1588, ν(C=N).

*(^Triphenyl^Iminopyridine)FeCl_2_* (**4a**) (light-orange solid, 0.46 g, 96**%)**: MALDI-TOF-MS (*m*/*z*): calcd. for C_25_H_20_ClFeN_2_: 439.0664, found: 439.0714 [M − Cl]^+^. Anal. calcd. for C_25_H_20_Cl_2_FeN_2_: C, 63.19; H, 4.24; N, 5.90; found: C, 62.88; H, 4.18; N, 5.67. IR/cm^−1^:1588, ν(C=N).

*(^dibenzhydryl^Iminopyridine)FeCl_2_* (**7a**) (burgundy solid, 0.61 g, 93%): MALDI-TOF-MS (*m*/*z*): calcd. for C_39_H_32_ClFeN_2_: 619.1603, found: 619.0020 [M − Cl]^+^. Anal. calcd. for C_39_H_32_Cl_2_FeN_2_: C, 71.47; H, 4.92; N, 4.27; found: C, 71.99; H, 4.87; N, 4.17. IR/cm^−1^:1593, ν(C=N).

### 2.4. General Procedure for the Synthesis of Cobalt Complexes

All complexes were prepared in a similar manner by the reaction of anhydrous CoCl_2_ with the corresponding ligands in tetrahydrofuran (THF). A typical synthetic procedure used for complexes **2b**, **3b**, and **5b** is as follows. Ligand (1.0 mmol) and CoCl_2_ (1.0 mmol) were stirred in 10 mL of THF overnight at room temperature. The precipitate was collected by filtration, washed with hexane (10 mL × 2) and dried under vacuum to obtain a blue or green solid.

*(^octyl^Iminopyridine)CoCl_2_* (**2b**) (blue solid, 0.30 g, 87%): MALDI-TOF-MS (*m*/*z*): calcd. for C1_4_H_22_ClCoN_2_: 312.0804, found: 311.9917 [M − Cl]^+^. Anal. calcd. for C1_4_H_22_Cl_2_CoN_2_: C, 48.30; H, 6.37; N, 8.05; found: C, 49.41; H, 6.45; N, 7.91. IR/cm^−1^:1597, ν(C=N).

*(^Adamantyl^Iminopyridine)CoCl_2_* (**3b**) (blue solid, 0.33 g, 90%): MALDI-TOF-MS (*m*/*z*): calcd. for C_16_H_20_ClCoN_2_: 334.0647, found: 333.9984 [M − Cl]^+^. Anal. calcd. for C_16_H_20_Cl_2_CoN_2_: C, 51.91; H, 5.45; N, 7.57; found: C, 52.03; H, 5.23; N, 7.88. IR/cm^−1^:1595, ν(C=N).

*(^supermesityl^Iminopyridine)CoCl_2_* (**5b**) (green solid, 0.49 g, 91%): MALDI-TOF-MS (*m*/*z*): calcd. for C_30_H_22_ClCoN_2_: 504.0804, found: 503.9194 [M − Cl]^+^. Anal. calcd. for C_30_H_22_Cl_2_CoN_2_: C, 66.68; H, 4.10; N, 5.18; found: C, 66.11; H, 3.96; N,5.31. IR/cm^−1^:1597, ν(C=N).

### 2.5. General Procedure for Isoprene Polymerization

The polymerization of isoprene in toluene was carried out in a 50 mL Schlenk reactor. In a typical experiment, the reactor was heated, dried in a vacuum, and recharged with nitrogen more than three times before the required amount of an aluminum coactivator, toluene (7 mL), and isoprene (2 mL) were added into the reactor. Then, 8.0 μmol of iron or cobalt complex in 1 mL CH_2_Cl_2_ was injected to initiate the polymerization at the desired temperature. After 2 h, the polymerization was quenched with a diluted HCl solution of methanol (methanol/HCl = 50/1). The polymer was collected by filtration and washed with ethanol several times and dried at room temperature for 24 h under vacuum.

### 2.6. Calculation of Microstructure Contents of Polyisoprenes

According to the calculated area of the characteristic signals at 4.66–4.72 and 5.12 ppm, the molar content of 3,4 units and 1,4 units based on ^1^H NMR spectra can be calculated by Equations (1) and (2) where *I* (5.12 ppm) and *I* (4.66–4.72 ppm) represent signal areas at 5.12 and 4.66–4.72 ppm.

(1) $$[\%1,4-\mathrm{units}]=\frac{I(5.12\;\mathrm{ppm})}{I(5.12\;\mathrm{ppm})+\frac{I(4.66-4.72\;\mathrm{ppm})}{2}}$$

(2) $$[\%3,4-\mathrm{units}]=\frac{\frac{I(4.66-4.72\;\mathrm{ppm})}{2}}{I(5.12\;\mathrm{ppm})+\frac{I(4.66-4.72\;\mathrm{ppm})}{2}}$$

According to the calculated area of the characteristic signals at 16.2 and 23.8 ppm, the molar content of *cis*-1,4 units and *trans*-1,4 units based on ^13^C NMR spectra can be calculated by Equations (3) and (4), where *I* (23.8 ppm) and *I* (16.2 ppm) represent signal areas at 23.8 and 16.2 ppm.

(3) $$[\%cis-1,4-\mathrm{units}]=\frac{I(23.8\;\mathrm{ppm})}{I(23.8\;\mathrm{ppm})\;+\;I(16.2\;\mathrm{ppm})}$$

(4) $$[\%trans-1,4-\mathrm{units}]=\frac{I(16.2\;\mathrm{ppm})}{I(23.8\;\mathrm{ppm})\;+\;I(16.2\;\mathrm{ppm})}$$

The microstructures of the polyisoprenes based on the FTIR spectra can be calculated according to the equations in the literature [50].

(5) $$A_{1375}=24[cis-1,4-\mathrm{units}]L+32.6[3,4-\mathrm{units}]L$$

(6) $$A_{890}=101[3,4-\mathrm{units}]L$$

(7) $$[\%cis-1,4-\mathrm{units}]=100\times \frac{\left[cis-1,4-\mathrm{units}\right]}{\left[cis-1,4-\mathrm{units}]\;+\;[3,4-\mathrm{units}\right]}$$

(8) $$[\%3,4-\mathrm{units}]=100\times \frac{\left[3,4-\mathrm{units}\right]}{\left[cis-1,4-\mathrm{units}]\;+\;[3,4-\mathrm{units}\right]}$$

where *A*_1375_ and *A*_890_ are the absorption intensity at 1375 and 890 cm^−1^, expressed by the peak height, [*cis*-1,4-units] represents the molar content of *cis*-1,4-units, [3,4-units] represents the molar content of 3,4-units, and *L* indicates the thickness of the sample.

## 3. Results and Discussion

### 3.1. Synthesis and Characterization of the Iron and Cobalt Complexes

**Scheme 2:**
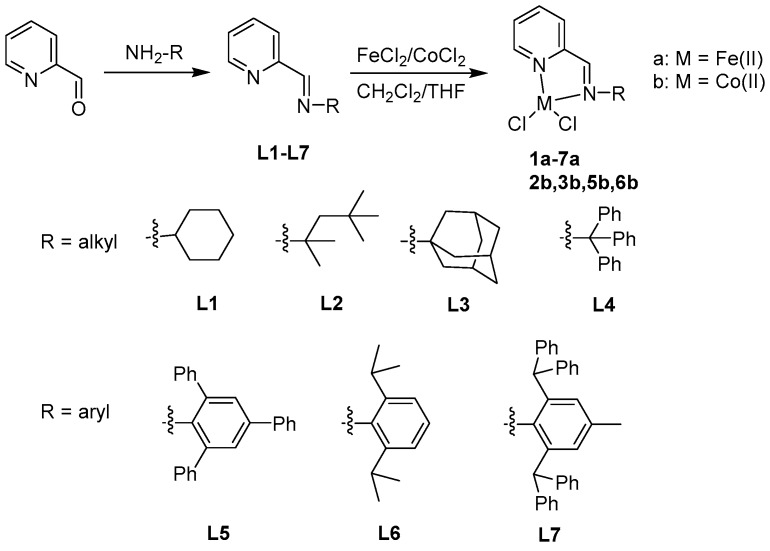
Synthesis of the ligands and the Fe(II) and Co(II) complexes.

The synthetic route for the iminopyridine complexes is shown in Scheme 2. The ligands were prepared at high yields by acid-catalyzed condensation between corresponding anilines and 2-pyridinecarboxaldehyde in methanol and identified by NMR (See Supplementary Materials, Figures S1–S6) and elemental analysis. The corresponding Fe(II) and Co(II) complexes (**1a**–**7a**, **2b**, **3b**, **5b**, **6b**) were prepared from the reaction of the ligands with 1 equiv of anhydrous FeCl_2_ or CoCl_2_ in CH_2_Cl_2_ and THF, respectively. These complexes were characterized by mass spectroscopy (See Supplementary Materials, Figures S7–S13) and elemental analysis.

**Figure 1:**
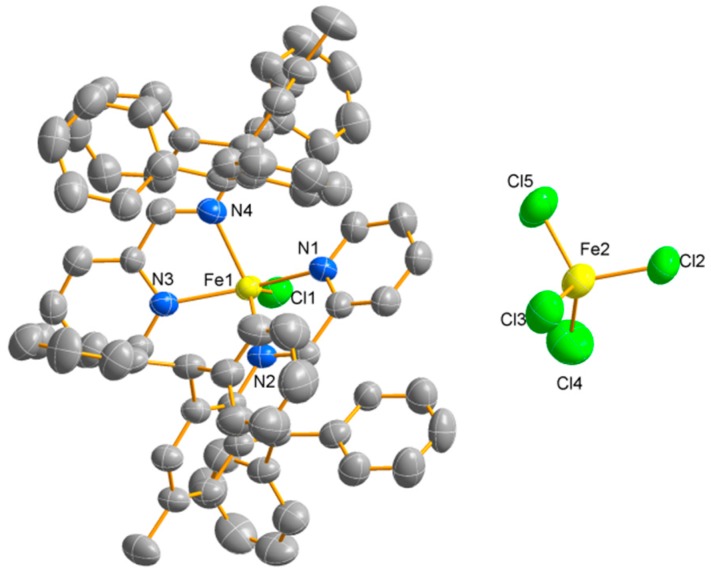
Molecular structure of complex **7a′**. (Thermal ellipsoids are shown at the 50% probability level.) Hydrogen atoms have been omitted for clarity.

**Figure 2:**
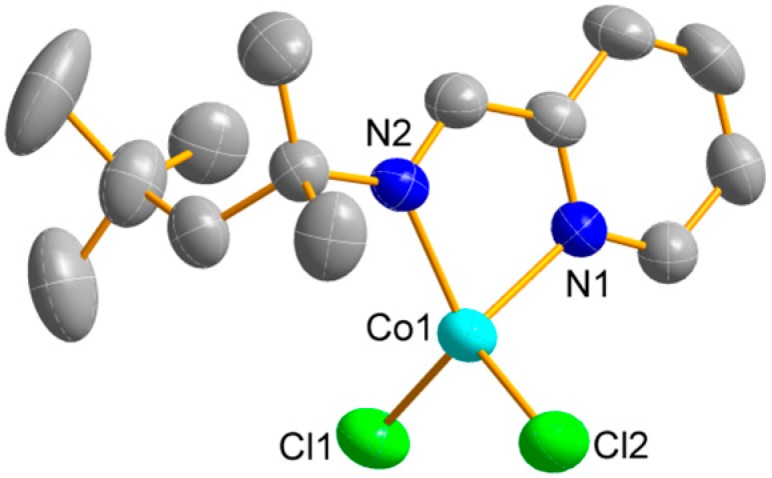
Molecular structure of complex **2b**. (Thermal ellipsoids are shown at the 50% probability level.) Hydrogen atoms have been omitted for clarity.

The structures of the complexes **1a**–**7a** should be those drawn in Scheme 2. This is supported by the elemental analysis, mass spectroscopy, and literature results on similar Fe(II) complexes [51]. Multiple attempts to grow single crystals of complexes **1a**–**7a** failed. However, during this process, single crystals of complex **7a′** were obtained and analyzed by X-ray diffraction (Figure 1, See Supplementary Materials, Tables S2 and S3). Complex **7a′** probably arises from the oxidation of **7a** during the recrystallization process. This unusual complex of **7a′** is interesting, and can prove the connectivity of the iminopyridine ligand to the metal center. The X-ray crystal structure analysis of **7a′** shows a distorted trigonal bipyramidal coordination geometry around the Fe(II) center. The steric environment of the ligand and the blocking of the axial position of the metal center from the dibenzhydryl moiety can be clearly observed from this molecular structure. Single crystals of pure complex **2b** could be obtained and the X-ray structure is shown in Figure 2. In a solid state, the cobalt center adopts a distorted tetrahedral coordination geometry with N1–Co–N2 angle of 81.61° and Cl1–Co–Cl2 angle of 112.06° (See Supplementary Materials, Tables S2 and S4). Complex **2b** shows shorter Co–N bond distance (2.040 and 2.046 Å) than aryl-substituted Co(II) complexes reported in literature [41] (2.044~2.181 Å), which may be attributed to the strong electron-donating effect of the octyl substituents.

### 3.2. Isoprene Polymerization Studies

#### 3.2.1. Polymerization of Isoprene with Iron Catalysts

The isoprene polymerization was evaluated using various common alkylaluminum reagents as cocatalysts. Triisobutylaluminum (TIBA) or AlEt_2_Cl cocatalysts were not effective at all. Both AlEtCl_2_ and MAO were able to activate **2a** for isoprene polymerization (Table 1, entries 1 and 2). However, the **2a**/MAO system can generate high molecular polyisoprenes. Therefore, MAO was chosen as the activator in the iminopyridine Fe(II) system (See Supplementary Materials, Table S1).

The alkyl and aryl moiety significantly influenced the catalytic performances of the complexes. The aryl-substituted complexes **5a**–**7a** produced polymers at higher yields (83.2%–98.1%) than the alkyl-substituted complexes **1a**–**4a** (58.2%–83.1%). The aryl moiety is electronically more withdrawing than the alkyl moiety, which can reduce the electron density on the metal center, leading to better monomer coordination and faster chain propagation. This is supported by the fact that complex **5a** bears the strongest electron-withdrawing substituent and displays the highest yield. In addition, the molecular weight of polyisoprenes obtained by aryl-substituted complexes **5a**–**7a** is higher than alkyl-substituted complexes **1a**–**4a** (10.3 × 10^4^~18.2 × 10^4^ vs. 6.0 × 10^4^~7.9 × 10^4^). Probably, the steric environment of the aryl moiety retards chain transfer reaction more effectively than the alkyl moiety (See Supplementary Materials, Figures S14–S20). This is supported by the fact that complex **7a** bears a sterically bulky dibenzhydryl-derived ligand framework and generates polyisoprene with the highest molecular weight (18.2 × 10^4^). The temperature influence on the catalytic performance was also investigated. Polymerization of isoprene at −25 °C showed lower yields (**2a**: 66.3% vs. 83.1%; **5a**: 81.0% vs. 98.1%) and afforded the polymer with higher molecular weight (**2a**: 7.9 × 10^4^ vs. 6.1 × 10^4^; **5a**: 15.4 × 10^4^ vs. 10.3 × 10^4^) than those at 25 °C.

**Figure 3:**
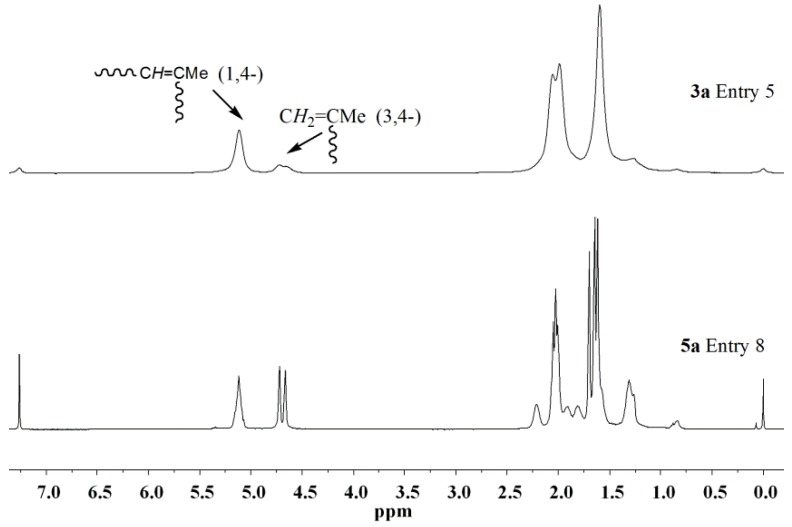
^1^H NMR spectra of polyisoprenes obtained by Fe(II) catalyst.

The microstructures of the resulting polyisoprenes were analyzed via ^1^H NMR and ^13^C NMR (See Supplementary Materials, Figures S23–S26) [51]. The representative ^1^H NMR spectra of the polyisoprenes obtained by the Fe(II) catalysts are shown in Figure 3. The 1,2-unit was not observed. The polyisoprene obtained by aryl-substituted complex **5a** contains 34.5% 3,4-units (Table 1, entry 10), which was much higher than that of the aryl-substituted complex **3a** (15.0%, entry 5). Similar trends were observed for other alkyl-substituted complexes (14.0%~15.0%) and aryl-substituted complexes (greater than 23.8%). Interestingly, the R group in the alkyl-substituted complexes only slightly influenced 3,4-selectivity from 14.0% to 15.0%. However, the selectivity of 3,4-units was increased from 23.8% to 34.5% when the steric hindrance of the aryl-substituted complexes was decreased. The high 3,4 units of polyisoprene can increase the toughness of the synthetic rubber and show outstanding wet skid resistance and low heat build-up when applied as car tires [55], thus representing a big advantage of this catalyst system. Additionally, the alkyl-substituted complexes **1a**–**4a** produced polymers with higher *cis*-1,4 content (77.1%~78.2%) than the aryl-substituted complexes **5a**–**7a** (62.7%–71.4%). At the same time, the alkyl-substituted complexes produced polyisoprene with 7.6%~8.9% *trans*-1,4 content, which was ca. twice as much as that by aryl-substituted complexes (2.8%~4.8%). However, polymers generated from the aryl-substituted complexes had the higher *cis*-1,4/*trans*-1,4 ratio (e.g., **5a**: 96:4, entry 8) than the alkyl-substituted complexes (e.g., **3a**: 90:10, entry 5). These results indicated that the electron-donating alkyl-substituted complexes tend to polymerize isoprene with *trans*-1,4-selectivity when 1,4-addition occurred. It was also observed that the steric hindrance of both kinds of complexes almost have minimum influence on *cis*-1,4/*trans*-1,4 stereoselectivity with **1a**–**4a** (ca. 90:10) and **5a**–**7a** (ca. 95:5).

Previously, Raynaud et al. used alkylaluminum/[Ph_3_C][B(C_6_F_5_)_4_] cocatalysts to activate the Fe(II) complexes, and the 1,4-*trans*/1,4-*cis* selectivity was affected by the alkyl/aryl substituents and the alkylaluminum agents (TIBA and AlEt_3_) [51]. In our system, the alkylaluminum/[Ph_3_C][B(C_6_F_5_)_4_] cocatalysts led to highly unreproducible results, which may originate from the high sensitivity of these Fe(II) complexes. As a result, MAO was chosen as the cocatalyst. These Fe(II) complexes showed high activities and high polymer molecular weight when activated using MAO as cocatalyst. Furthermore, in our Fe(II)/MAO system, the aryl-substituted iminopyridine iron complexes also favor 3,4-insertion and give rise to higher amounts of 3,4-units than the alkyl-substituted iminopyridine iron complexes, which is similar to the Fe(II)/alkylaluminum/[Ph_3_C][B(C_6_F_5_)_4_] system. However, there are some notable differences between these two systems. In our Fe(II)/MAO system, the ratio between 1,4-*cis*/*trans* units was not affected by the aryl or alkyl substituents. Although this difference is not fully understood, it is clear that the cocatalysts may play an important role in determining the stereoselectivity.

#### 3.2.2. Polymerization of Isoprene with Co(II) Catalysts

The polymerization results using Co(II) complexes **2b**, **3b**, **5b**, and **6b** are summarized in Table 2. Four cocatalysts (TIBA, AlEt_2_Cl, AlEtCl_2_, and MAO) were used in attempts to generate the active catalysts. Only cocatalyst AlEtCl_2_ was able to activate Co(II) complex **2b** for isoprene polymerization. Although the yields (greater than 76.9%) of polyisoprene generated from Co(II) complexes are similar with those of Fe(II) complexes, there are some apparent differences between the Co(II) and the Fe(II) systems. In sharp contrast to the Fe(II) complexes, the polymers produced by Co(II) complexes were white powder with molecular weights below 2000 and broad molecular distribution of above 4.76 (See Supplementary Materials, Figures S21 and S22). Moreover, complexes **5b** and **6b** containing electron-withdrawing aryl substituents afforded polymers with higher molecular weights (**5b**: 1700, **6b**: 1800) at higher yields (**5b**: 97.3%, **6b**: 94.9%) than those by complexes **2b** and **3b** containing electron-donating alkyl substituents (**2b**: 1400 and 78.2%, **3b**: 1500 and 76.9%). This is similar with the trend observed in the Fe(II) systems.

**Figure 4:**
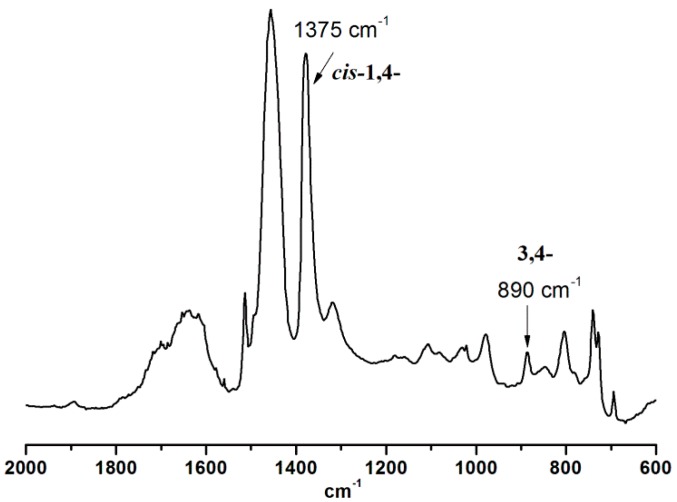
FTIR spectra of polyisoprenes obtained by Co(II) catalysts (entry 6).

The ^1^H NMR and ^13^C NMR spectra of polyisoprene obtained by Co(II) complexes have the broad peaks and low resolution because of the low molecular weight of the polymers (See Supplementary Materials, Figures S27–S30). It was difficult to assign the peaks of these polymers in the ^1^H NMR and ^13^C NMR spectra, so FTIR measurements were carried out to determine and analyze the microstructures of the polyisoprenes (See Supplementary Materials, Figures S31–S34). The absorption bands at 1375 and 890 cm^−1^ correspond to the *cis*-1,4 and the 3,4-units [50]. The typical bands of *trans*-1,4 units are at 845, 1152, 1325, and 1385 cm^−1^ and the band of 1,2-units is at 911 cm^−1^ [50]. As shown in Figure 4, no bands were observed for the *trans*-1,4 unit or 1,2-unit in the spectrum. Based on the equations shown in the experimental section, the polymer generated with the Co(II)/AlEtCl_2_ system is composed of predominantly *cis*-1,4 units (ca. 90%) along with a small amount of 3,4-units (ca. 10%). Interestingly, the Co(II) system produced polymers with higher *cis*-1,4 content (ca. 90%) than the Fe(II) system (65%~85%). The stereoregularity of the polyisoprenes was only slightly influenced by the ligand environment.

## 4. Conclusions

In conclusion, a series of iminopyridine Fe(II) and Co(II) complexes bearing various alkyl and aryl substituents was prepared. The aim is to systematically investigate the influence of alkyl and aryl substituents on the isoprene polymerization. Activated by MAO, the Fe(II) complexes exhibited moderate *cis*-1,4 selectivity, generating high molecular weight polyisoprenes. The Fe(II) catalyzed polymerization of isoprene was relatively sensitive to alkyl and aryl substituents. High 3,4-units (up to 34.5%) and high molecular weight (10.3 × 10^4^~18.2 × 10^4^) polyisoprenes can be obtained using aryl-substituted Fe(II) complexes. Meanwhile, the Co(II)/AlEt_2_Cl system exhibited relatively high *cis*-1,4-selectivity, affording low molecular weight polyisoprenes. The alkyl and aryl substituents in Co(II) complexes did not significantly influence the selectivity and molecular weight of the resulting polymers.

## Figures and Tables

**Table 1:** Isoprene polymerization results with Fe(II) catalyst ^a^.

Entry	Complex	*T* (°C)	Yield (%)	Activity ^c^	*M*_n_^d^ (×10^−4^)	PDI ^d^	Microstructure ^c^ (%) ^e^			
							*cis*-1,4	*trans*-1,4	*cis*/*trans*	3,4
1 ^b^	**2a**	25	83.4	7.1	0.18	4.70	-	-		-
2	**2a**	25	83.1	7.1	6.1	1.57	77.5	8.1	91:9	14.4
3	**2a**	−25	66.3	5.6	7.9	2.45	77.0	8.7	90:10	14.3
4	**1a**	25	64.1	5.4	6.0	2.11	77.1	8.9	90:10	14.0
5	**3a**	25	58.2	4.9	7.0	1.82	76.8	8.2	90:10	15.0
6	**4a**	25	61.3	5.2	6.1	2.08	78.2	7.6	91:9	14.2
7	**5a**	−25	81.0	6.9	15.4	2.13	63.9	3.0	96:4	33.1
8	**5a**	25	98.1	8.3	10.3	2.05	62.7	2.8	96:4	34.5
9	**6a**	25	83.2	7.1	18.0	1.75	69.9	4.5	94:6	25.6
10	**7a**	25	85.7	7.3	18.2	1.61	71.4	4.8	94:6	23.8

^a^ Polymerization conditions: 8.0 μmol of Fe(II) complex; MAO/Fe = 500; 7 mL toluene and 1 mL CH_2_Cl_2_; isoprene = 2 mL; time = 2 h; ^b^ activator = AlEtCl_2_, Al/Fe = 150; ^c^ 10^4^ g of polyisorene (mol of Fe)^−1^·h^−1^; ^d^ determined by gel permeation chromatography (GPC); ^e^ determined by ^1^H NMR and ^13^C NMR.

**Table 2:** Isoprene polymerization results with Co(II) catalyst ^a^.

Entry	Complex	Yield (%)	Activity ^b^	*M*_n_^c^ (×10^−3^)	PDI ^c^	Microstructure ^d^ (%)	
						*cis*-1,4	3,4
1	**2b**	78.2	6.6	1.4	7.97	91.1	8.9
2	**3b**	76.9	6.5	1.5	4.76	90.8	9.2
3	**5b**	97.3	8.3	1.7	8.05	88.1	11.9
4	**6b**	94.9	8.1	1.8	9.38	89.7	10.3

^a^ Polymerization conditions: 8.0 μmol of Co(II) complex; activator = AlEtCl_2_, Al/Co = 150; 7 mL toluene and 1 mL CH_2_Cl_2_; isoprene = 2 mL; time = 2 h; ^b^ 10^4^ g of polyisorene (mol of Co)^−1^·h^−1^; ^c^ determined by GPC; ^d^ determined by Fourier-transform infrared (FTIR) spectroscopy.

## Supplementary Materials

The following are available online at www.mdpi.com/2073-4360/8/11/389/s1. Optimization of MAO/Fe Ratio with **3a** (Table S1), NMR spectra of the ligands **L1**, **L3** and **L4** (Figures S1–S6), MALDI-TOF-MS of complexes (Figures S7–S13), crystal data of complex **7a′** (CCDC number: 1503575) and **2b** (CCDC number: 1503576) (Tables S2–S4), GPC curves of polyisoprene samples (Figures S14–S22), NMR spectra of the representive polyisoprenes (Figures S23–S30) and FTIR spectra of representive polyisoprenes (Figures S31–S34).
