# Supplementary material for: Influences of Alkyl and Aryl Substituents on Iminopyridine Fe(II)- and Co(II)-Catalyzed Isoprene Polymerization
Source: Polymers (Basel). 2016 Nov 3;8(11):389. doi: 10.3390/polym8110389 (PMC6432129; doi:10.3390/polym8110389)
Supplement: Supplementary file 1 [file polymers-08-00389-s001.pdf]

Supplementary Materials: Influences of Alkyl and Aryl Substituents on Iminopyridine Fe(II) and Co(II) Catalyzed Isoprene Polymerization

Lihua Guo, Xinyu Jing, Shuoyan Xiong, Wenjing Liu, Yanlan liu, Zhe Liu and Changle Chen

1. Optimization of MAO/Fe Ratio with 3a

Table S1. Optimization of MAO/Fe ratio with **3a** [a].

Entry	Al/Fe	Yield (%)	Activity [b]	M_n [c] ($\times 10^{-4}$)	PDI [c]
1	200	12.1	1.0	3.1	2.63
2	500	58.2	4.9	7.0	1.82
3	800	40.1	3.4	1.3	2.07

[a] Polymerization conditions: 8.0 μmol of Fe(II) complex; Reaction temperature = 25 °C, 7 mL toluene and 1 mL CH_2Cl_2; isoprene = 2 mL; time = 2 h; [b] 10^4 g of Polyisorene (mol of Fe)$^{-1}\cdot$h^{-1}; [c] Determined by GPC.

Catalytic activity increased when the Al/Fe ratio was increased from 200:1 to 500:1, but activity decreased when Al/Fe ratio was further increased to 800:1.

2. ^{1}H NMR, ^{13}C NMR of the Ligands

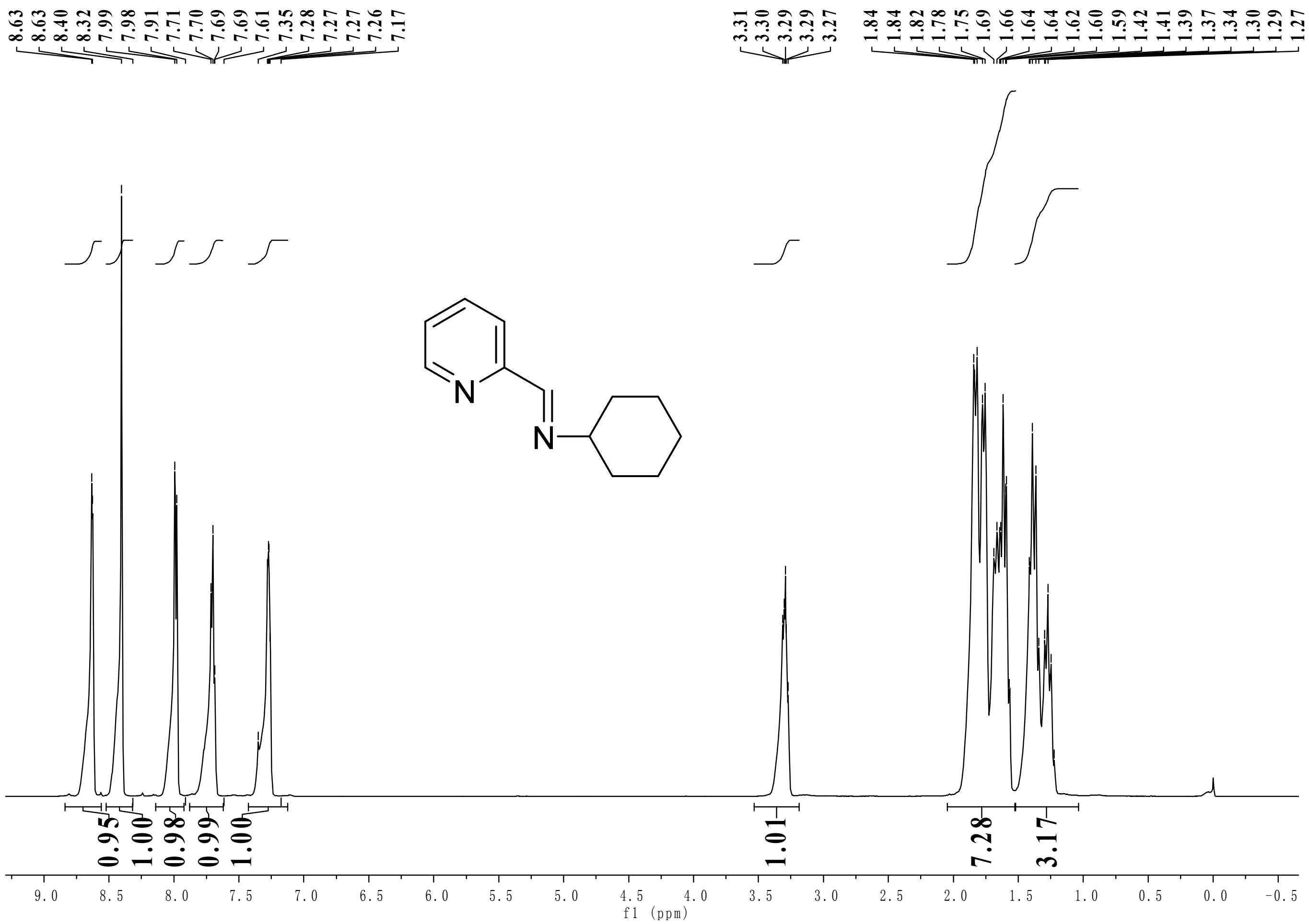

Figure S1. ^{1}H NMR spectrum (500 MHz) of **L1** in $CDCl_3$.

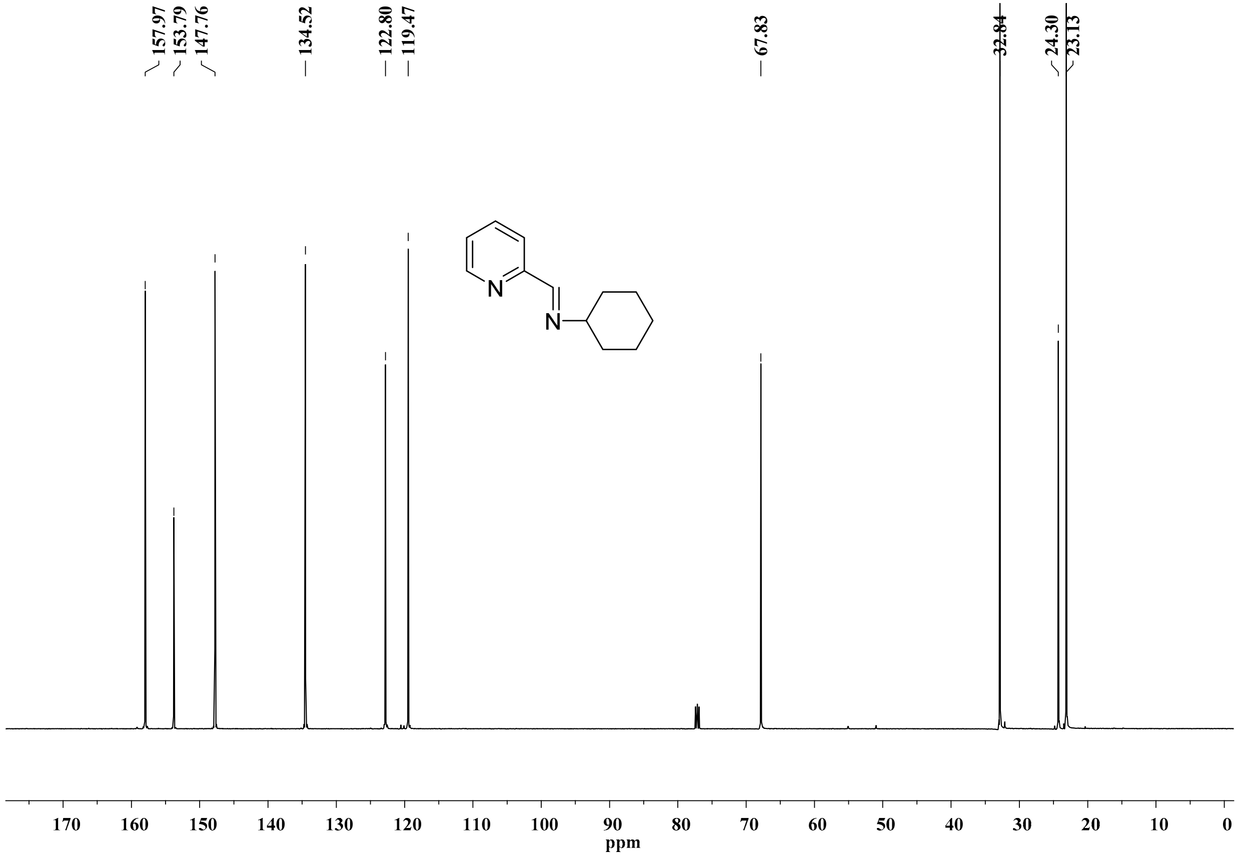

Figure S2. ^{13}C NMR spectrum (126 MHz) of **L1** in $CDCl_3$.

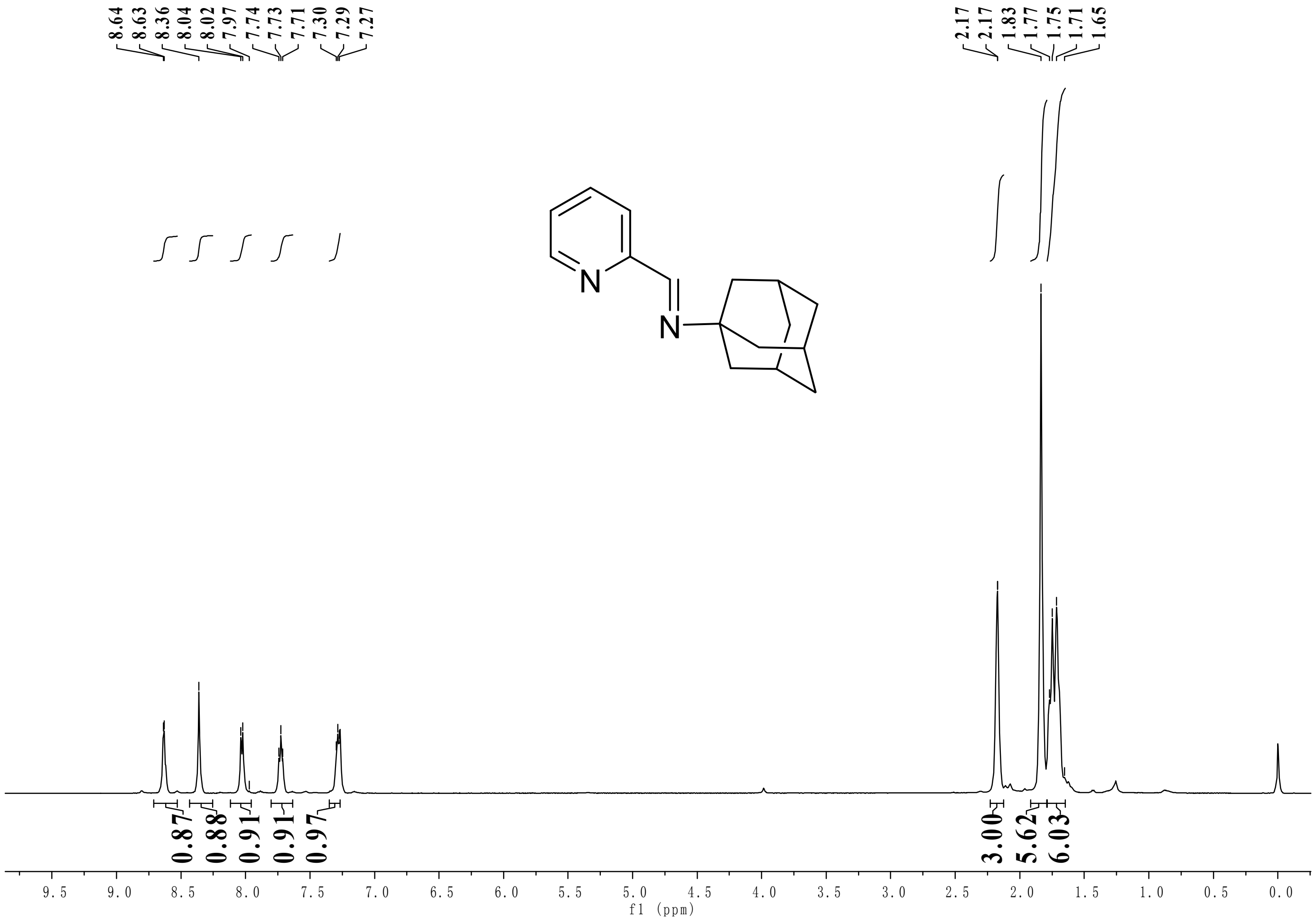

Figure S3. ^{1}H NMR spectrum (500 MHz) of **L3** in $CDCl_3$.

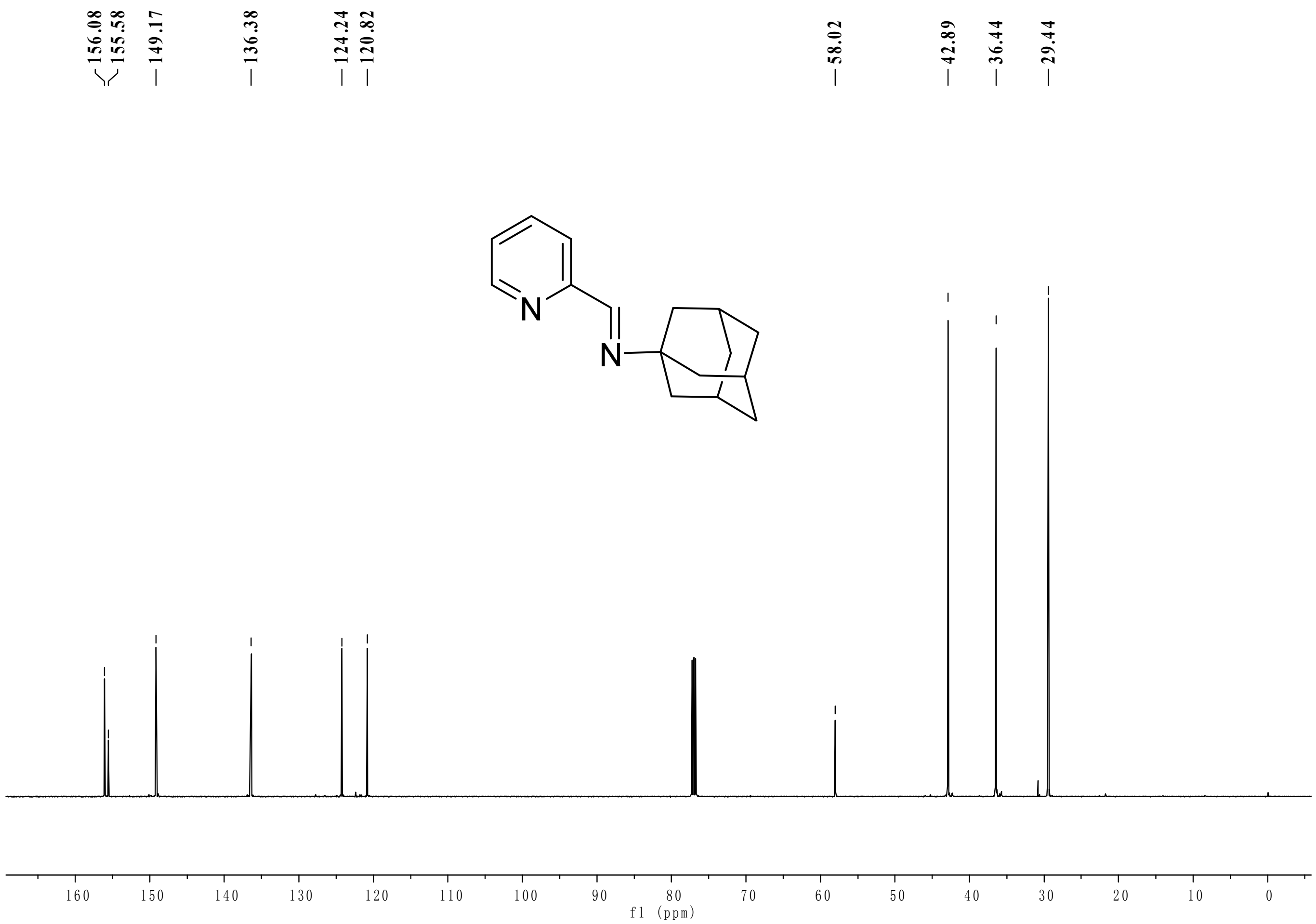

Figure S4. ^{13}C NMR spectrum (126 MHz) of **L3** in $CDCl_3$.

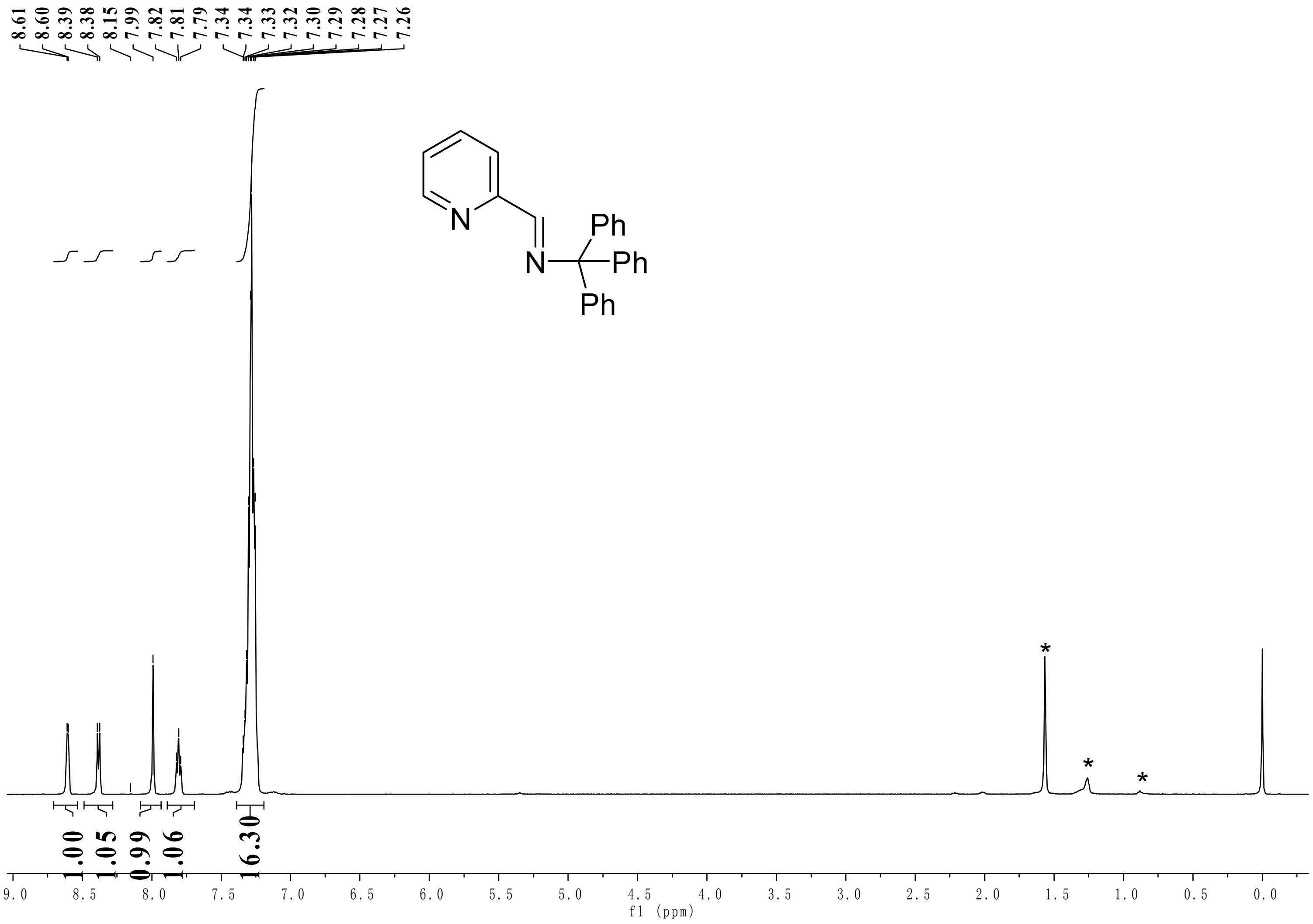

Figure S5. ^{1}H NMR spectrum (500 MHz) of **L4** in $CDCl_3$. * Hexane, H_2O.

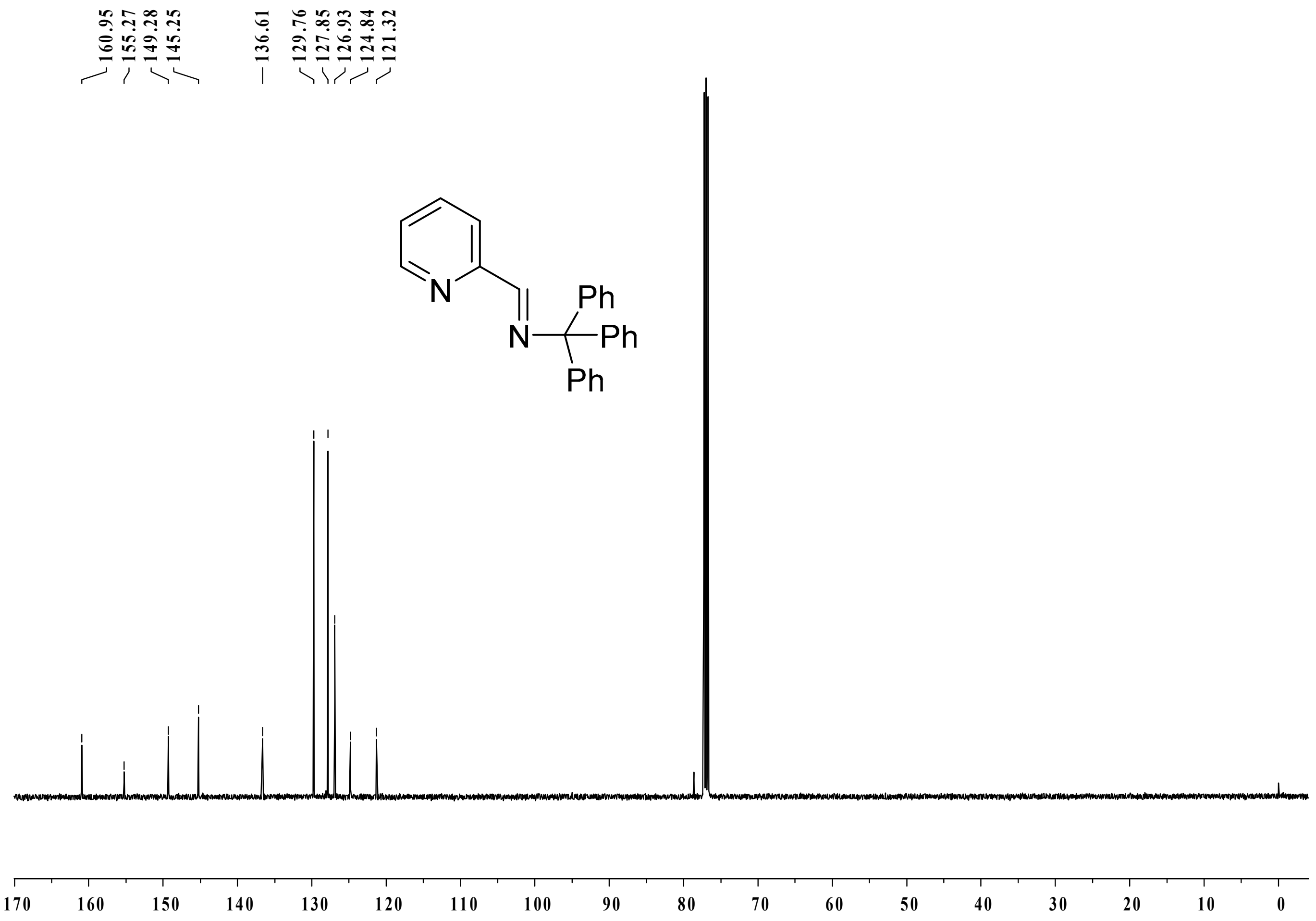

Figure S6. ^{13}C NMR spectrum (126 MHz) of **L4** in $CDCl_3$.

3. MALDI-TOF-MS of Fe(II) and Co(II) Complexes

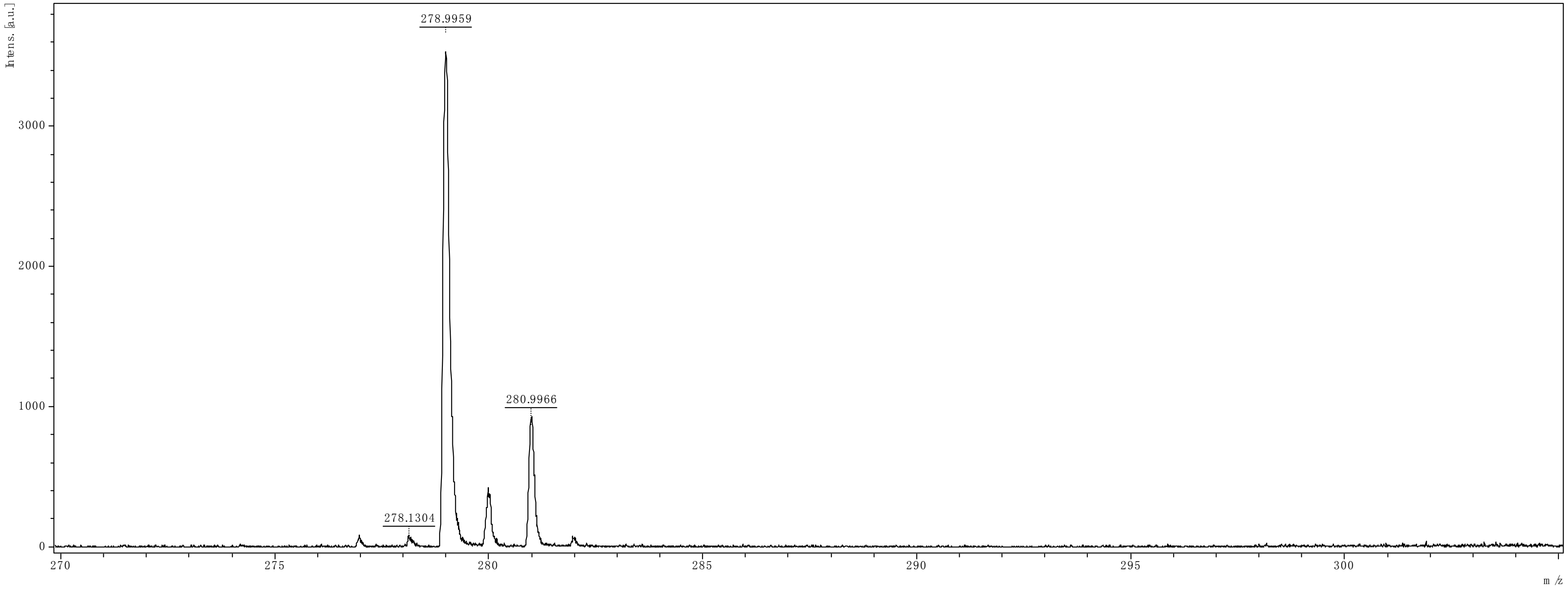

Figure S7. MALDI-TOF-MS of complex **1a**.

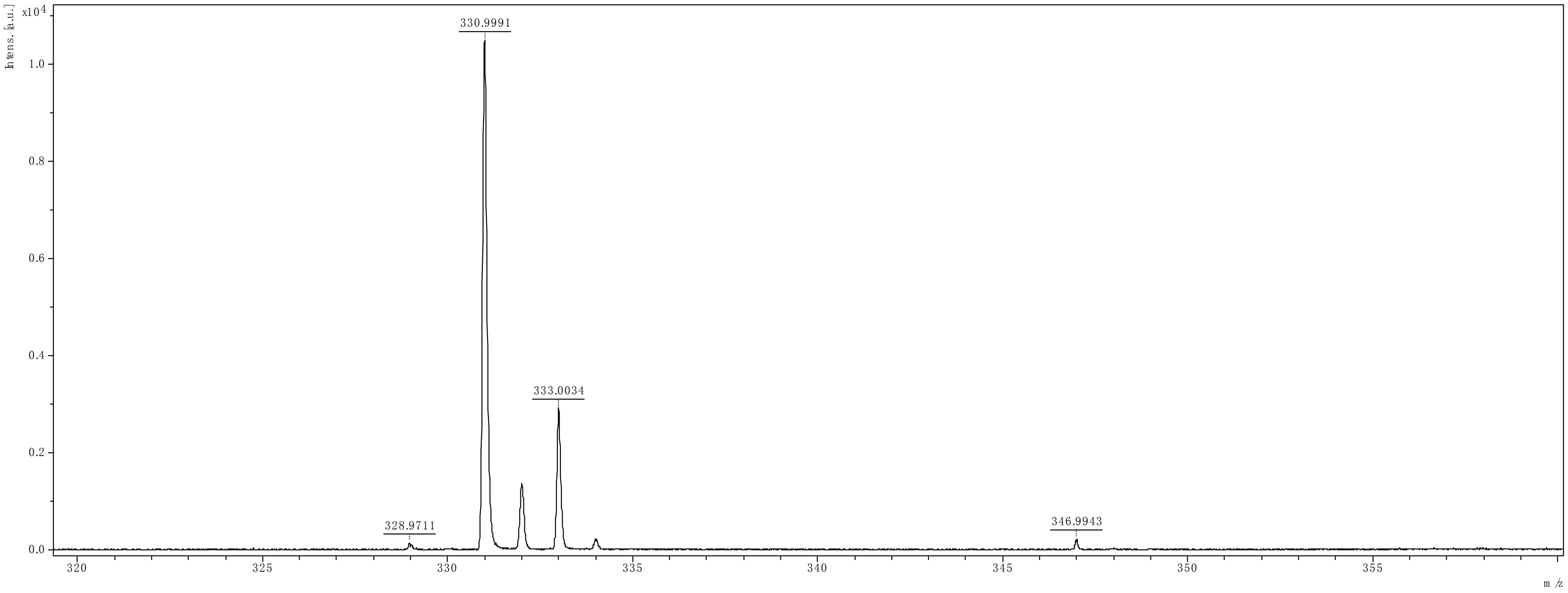

Figure S8. MALDI-TOF-MS of complex **3a**.

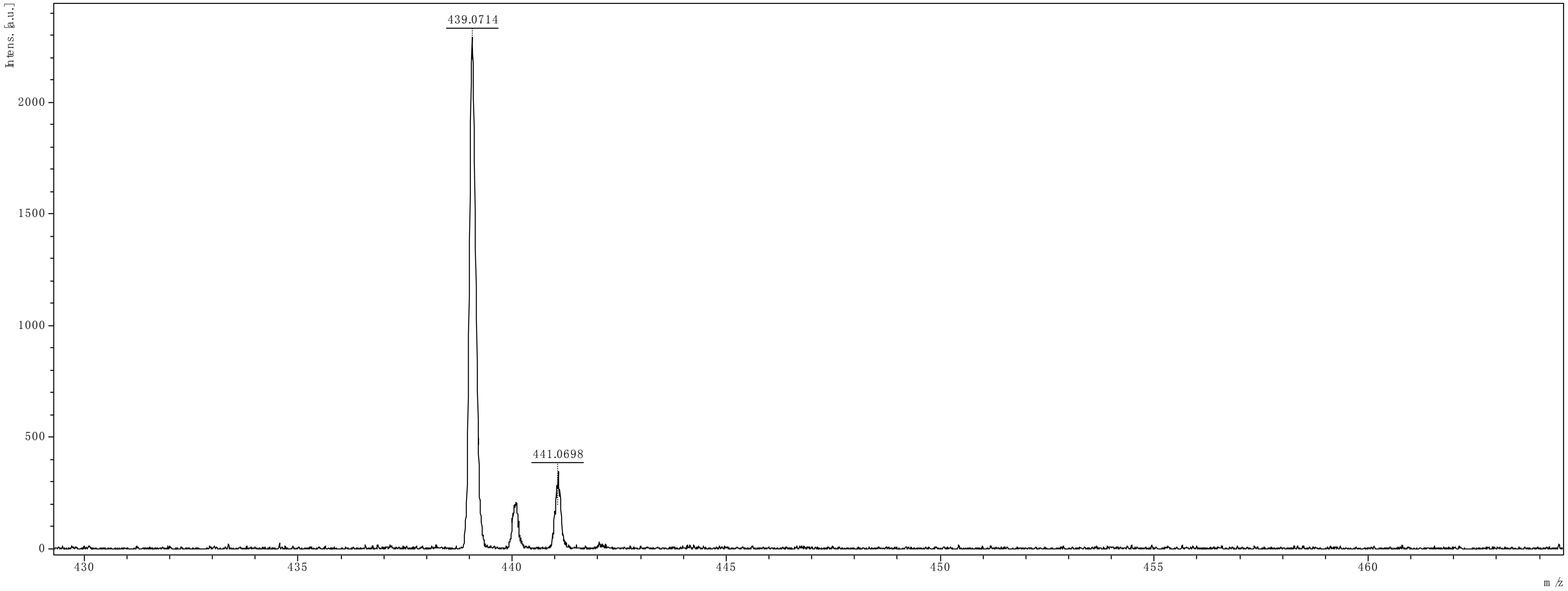

Figure S9. MALDI-TOF-MS of complex **4a**.

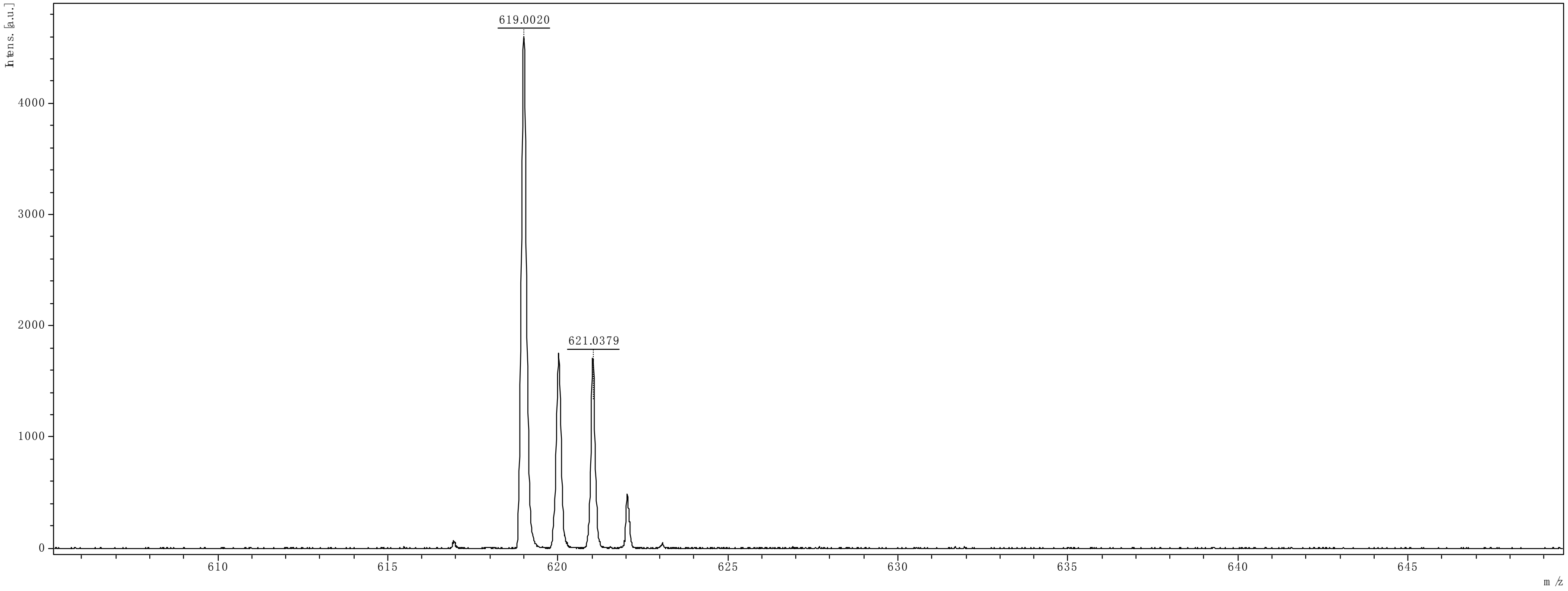

Figure S10. MALDI-TOF-MS of complex **7a**.

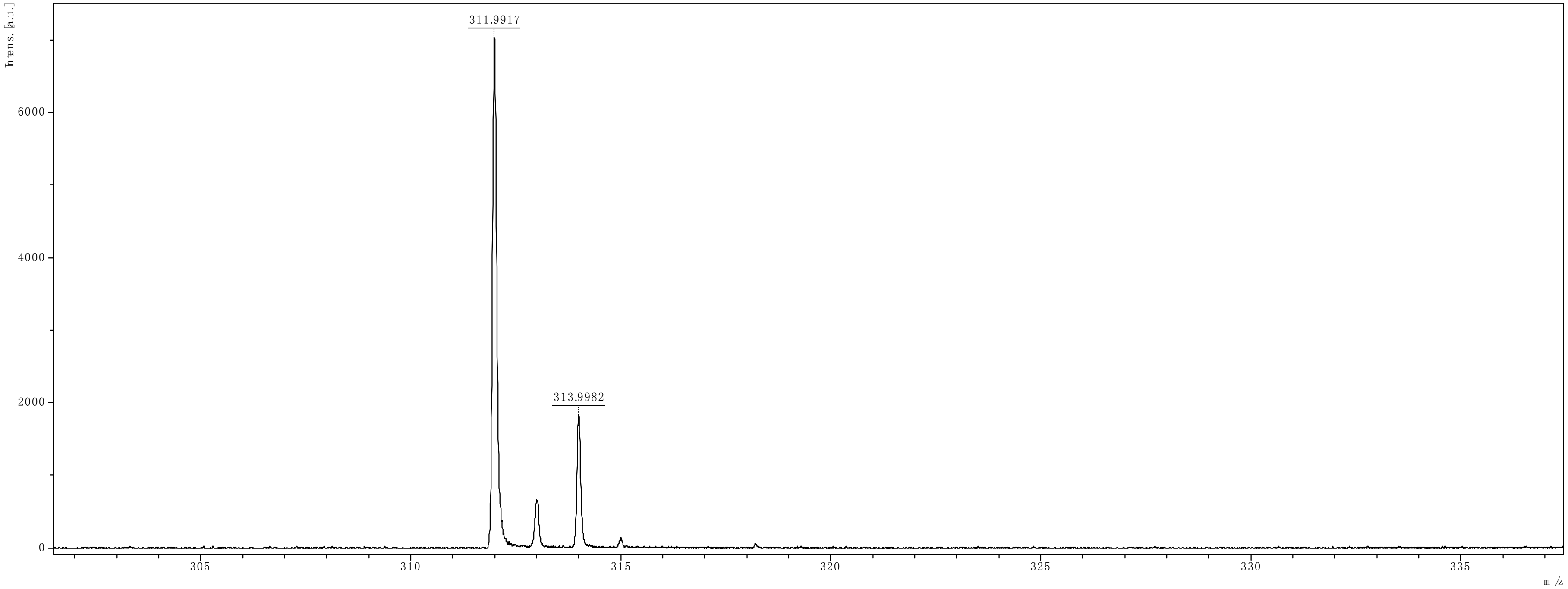

Figure S11. MALDI-TOF-MS of complex **2b**.

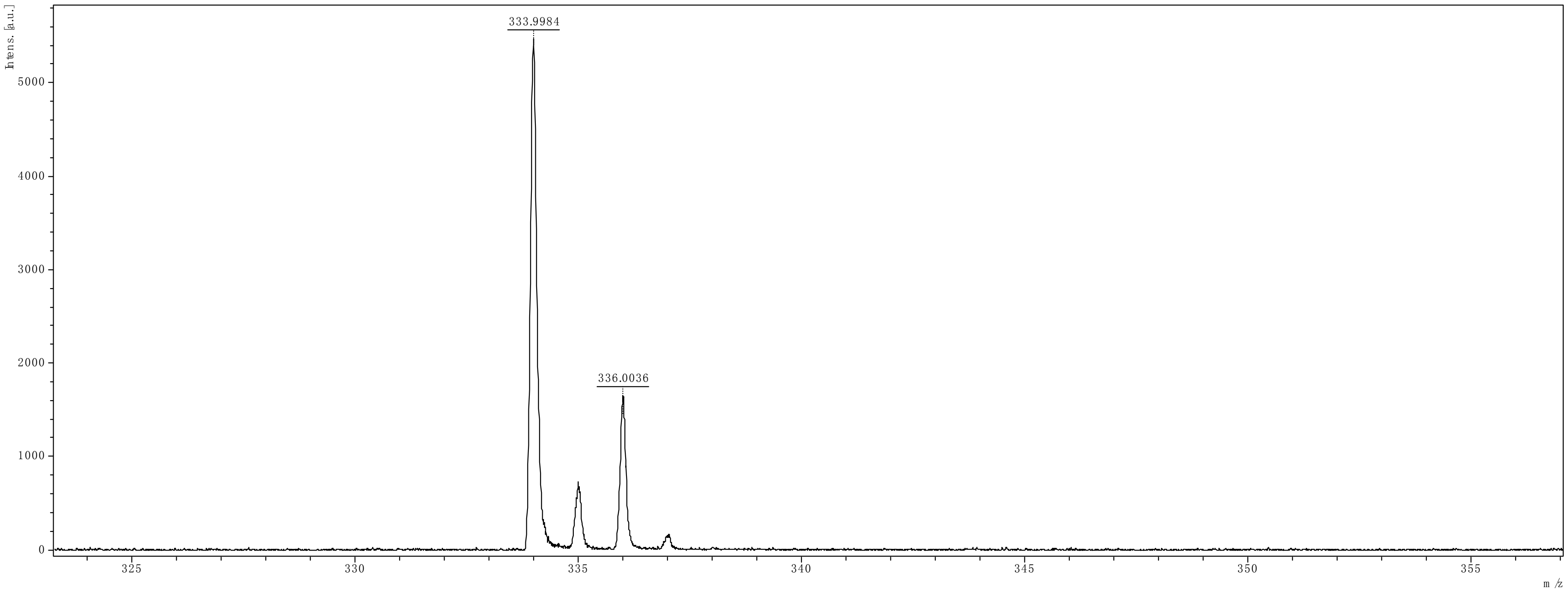

Figure S12. MALDI-TOF-MS of complex **3b**.

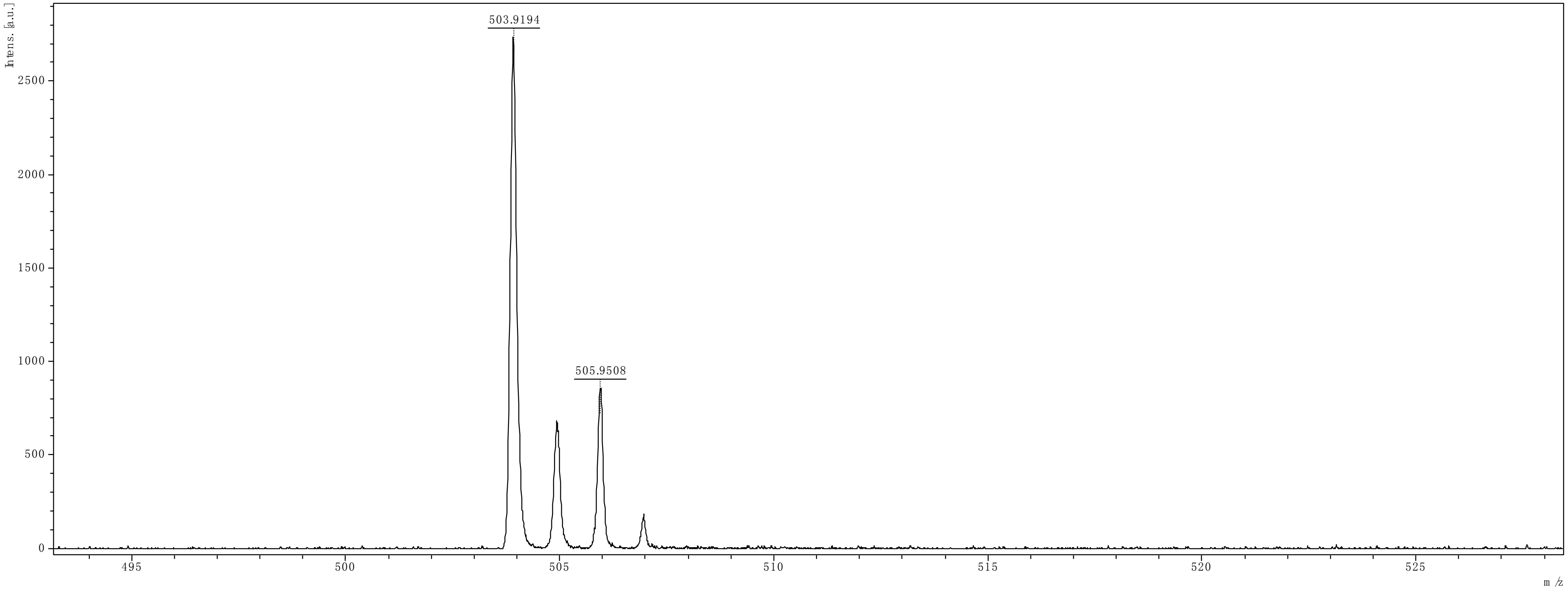

Figure S13. MALDI-TOF-MS of complex **5b**.

4. GPC Curves of Representive Polyisoprene Samples

Project Name Test
Reported by User: Breeze user (Breeze)

Breeze 2 HPLC System

SAMPLE INFORMATION

Sample Name:	JXY-K3	Acquired By:	Breeze
Sample Type:		Date Acquired:	2015/9/25 9:09:44 CST
Vial:	1	Acq. Method:	GPC
Injection #:	39	Date Processed:	2015/9/25 9:47:24 CST
Injection Volume:	20.00 ul	Channel Name:	410
Run Time:	34.00 Minutes	Sample Set Name	

Autoscaled Chromatogram

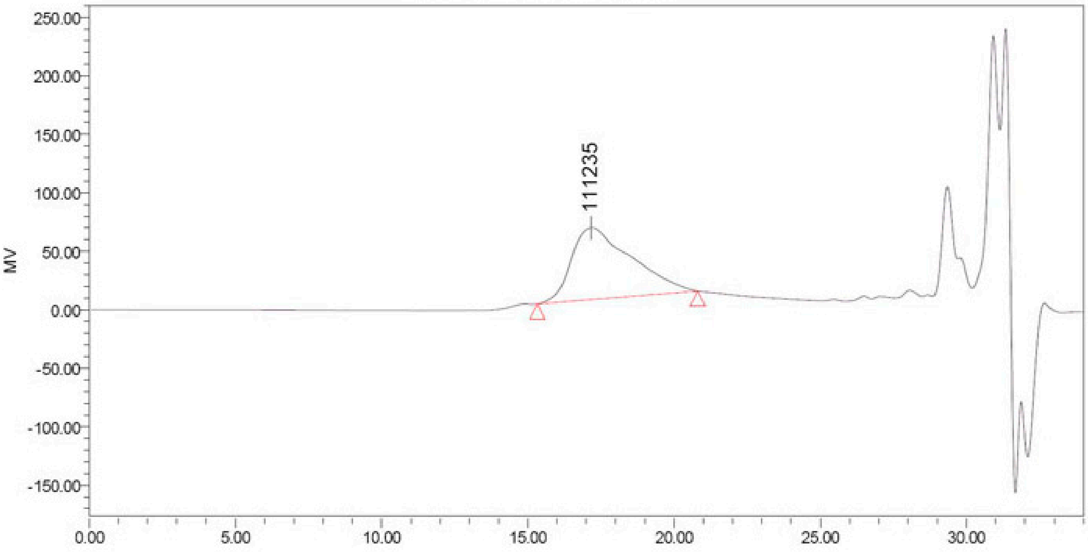

GPC Results

	Dist Name	Elution Volume (ml)	Retention Time (min)	Adjusted RT (min)	Mn	Mw	MP	Mz	Mz+1	Mz/Mw	Mz+1/Mw
1		17.167	17.167	17.167	60605	94854	11123[illegible]	134518	174152	1.418158	1.835995

GPC Results

	Area (*sec)	% Area	Height	% Height	Integration Type	Peak Codes	Points Across Peak	Start Time (min)	End Time (min)	Baseline Start (min)
1	8716480	100.00	61474	100.00	Bb	I08	658	15.325	20.808	15.325

Figure S14. GPC curves of Polyisoprene (Table 1, Entry 2).

Project Name Test
Reported by User: Breeze user (Breeze)

Breeze 2 HPLC System

SAMPLE INFORMATION

Sample Name:	JXY-L2	Acquired By:	Breeze
Sample Type:		Date Acquired:	2015/9/25 9:46:30 CST
Vial:	1	Acq. Method:	GPC
Injection #:	40	Date Processed:	2015/9/25 11:01:16 CST
Injection Volume:	20.00 ul	Channel Name:	410
Run Time:	34.00 Minutes	Sample Set Name	

Autoscaled Chromatogram

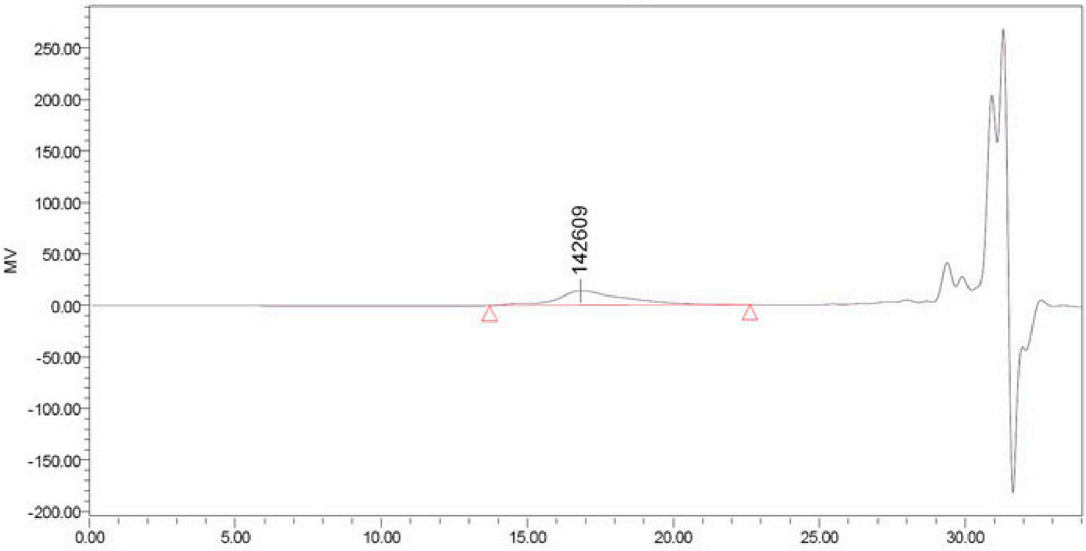

GPC Results

	Dist Name	Elution Volume (ml)	Retention Time (min)	Adjusted RT (min)	Mn	Mw	MP	Mz	Mz+1	Mz/Mw
1		16.835	16.835	16.835	60168	126854	142609	213692	307500	1.684549

GPC Results

	Mz+1/Mw	Area (*sec)	% Area	Height	% Height	Integration Type	Peak Codes	Points Across Peak	Start Time (min)	End Time (min)
1	2.424049	2319917	100.00	14189	100.00	bb		1070	13.708	22.625

Figure S15. GPC curves of Polyisoprene (Table 1, Entry 4).

Project Name Test
Reported by User: Breeze user (Breeze)

Breeze 2 HPLC System

SAMPLE INFORMATION

Sample Name:	JXY-k12	Acquired By:	Breeze
Sample Type:		Date Acquired:	2015/9/25 10:24:55 CST
Vial:	1	Acq. Method:	GPC
Injection #:	41	Date Processed:	2015/9/25 11:01:45 CST
Injection Volume:	20.00 ul	Channel Name:	410
Run Time:	34.00 Minutes	Sample Set Name	

Autoscaled Chromatogram

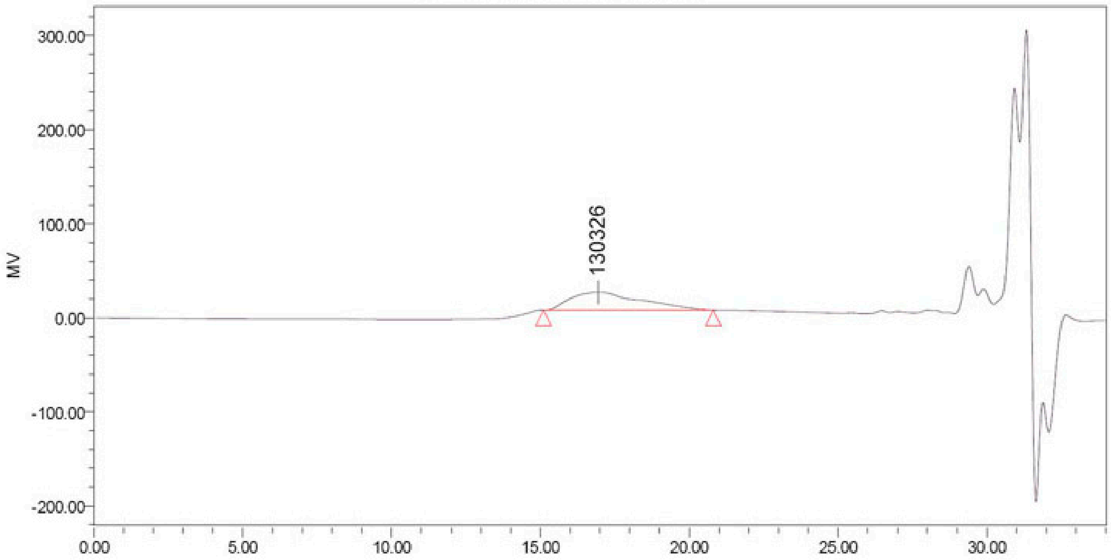

GPC Results

	Dist Name	Elution Volume (ml)	Retention Time (min)	Adjusted RT (min)	Mn	Mw	MP	Mz	Mz+1	Mz/Mw
1		16.953	16.953	16.953	69752	127031	130326	196190	256639	1.544431

GPC Results

	Mz+1/Mw	Area (*sec)	% Area	Height	% Height	Integration Type	Peak Codes	Points Across Peak	Start Time (min)	End Time (min)
1	2.020290	3190799	100.00	19143	100.00	Bb		684	15.108	20.808

Figure S16. GPC curves of Polyisoprene (Table 1, Entry 5).

Project Name Test
Reported by User: Breeze user (Breeze)

SAMPLE INFORMATION

Sample Name:	JXY-L7	Acquired By:	Breeze
Sample Type:		Date Acquired:	2015/9/24 9:49:07 CST
Vial:	1	Acq. Method:	GPC
Injection #:	35	Date Processed:	2015/9/24 16:46:18 CST
Injection Volume:	20.00 ul	Channel Name:	410
Run Time:	34.00 Minutes	Sample Set Name	

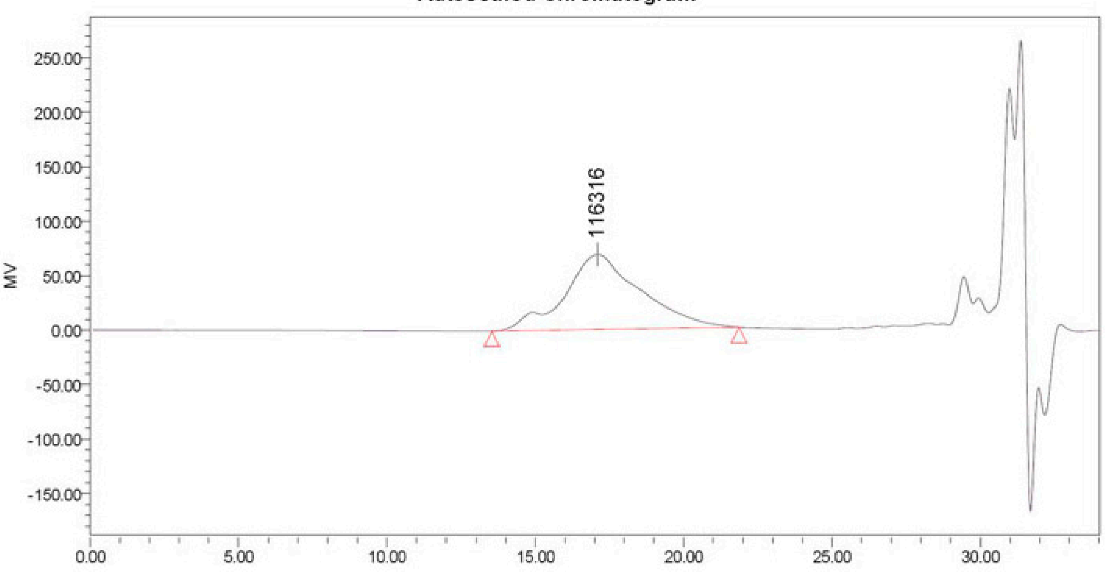

GPC Results

	Dist Name	Elution Volume (ml)	Retention Time (min)	Adjusted RT (min)	Mn	Mw	MP	Mz	Mz+1	Mz/Mw
1		17.106	17.106	17.106	61036	127382	11631€	227314	334456	1.784509

GPC Results

	Mz+1/Mw	Area (*sec)	% Area	Height	% Height	Integration Type	Peak Codes	Points Across Peak	Start Time (min)	End Time (min)
1	2.625612	12263850	100.00	68783	100.00	BB		999	13.542	21.867

Figure S17. GPC curves of Polyisoprene (Table 1, Entry 6).

Project Name Test
Reported by User: Breeze user (Breeze)

Breeze 2 HPLC System

SAMPLE INFORMATION

Sample Name:	JXY-X	Acquired By:	Breeze
Sample Type:		Date Acquired:	2015/9/24 15:26:20 CST
Vial:	1	Acq. Method:	GPC
Injection #:	37	Date Processed:	2015/9/24 16:47:02 CST
Injection Volume:	20.00 ul	Channel Name:	410
Run Time:	34.00 Minutes	Sample Set Name	

Autoscaled Chromatogram

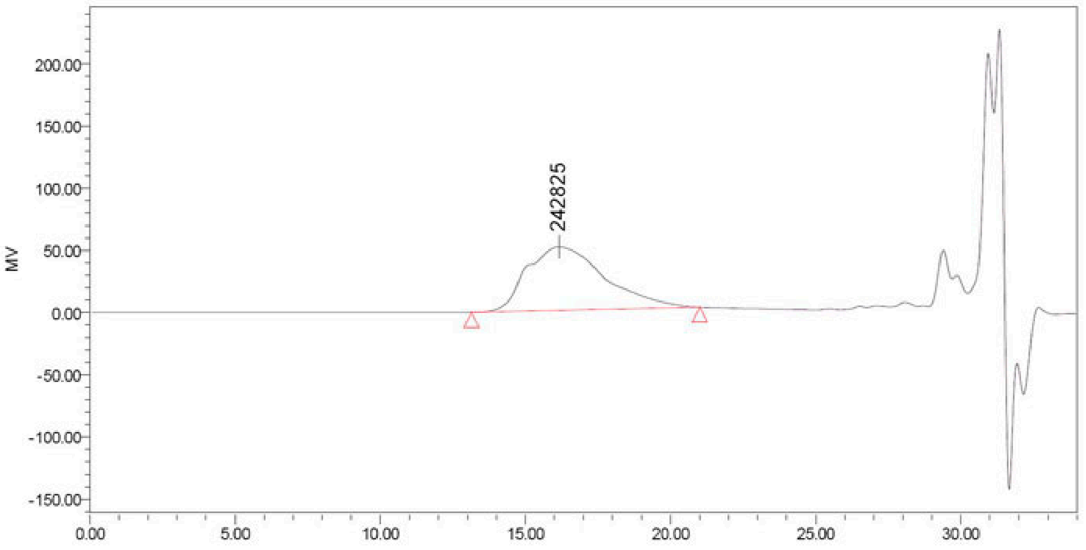

GPC Results

	Dist Name	Elution Volume (ml)	Retention Time (min)	Adjusted RT (min)	Mn	Mw	MP	Mz	Mz+1	Mz/Mw
1		16.166	16.166	16.166	102511	209808	242825	323257	406724	1.540730

GPC Results

	Mz+1/Mw	Area (*sec)	% Area	Height	% Height	Integration Type	Peak Codes	Points Across Peak	Start Time (min)	End Time (min)
1	1.938558	9572152	100.00	51012	100.00	Bb		944	13.142	21.008

Figure S18. GPC curves of Polyisoprene (Table 1, Entry 8).

Project Name Test
Reported by User: Breeze user (Breeze)

Breeze 2 HPLC System

SAMPLE INFORMATION

Sample Name:	JXY-S1	Acquired By:	Breeze
Sample Type:		Date Acquired:	2015/9/24 9:11:42 CST
Vial:	1	Acq. Method:	GPC
Injection #:	34	Date Processed:	2015/9/25 9:48:16 CST
Injection Volume:	20.00 ul	Channel Name:	410
Run Time:	34.00 Minutes	Sample Set Name	

Autoscaled Chromatogram

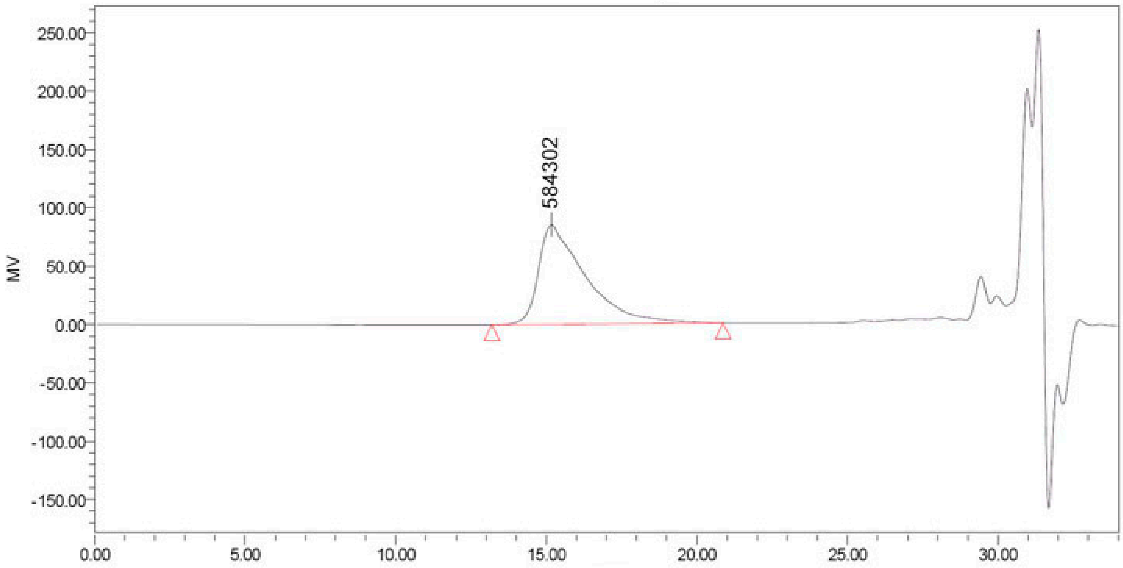

GPC Results

	Dist Name	Elution Volume (ml)	Retention Time (min)	Adjusted RT (min)	Mn	Mw	MP	Mz	Mz+1	Mz/Mw
1		15.175	15.175	15.175	179952	315274	584302	406477	461988	1.289280

GPC Results

	Mz+1/Mw	Area (*sec)	% Area	Height	% Height	Integration Type	Peak Codes	Points Across Peak	Start Time (min)	End Time (min)
1	1.465353	9290756	100.00	85464	100.00	Bb		921	13.192	20.867

Figure S19. GPC curves of Polyisoprene (Table 1, Entry 9).

Project Name Test
Reported by User: Breeze user (Breeze)

Breeze 2 HPLC System

SAMPLE INFORMATION

Sample Name:	JXY-S2	Acquired By:	Breeze
Sample Type:		Date Acquired:	2015/9/24 14:45:23 CST
Vial:	1	Acq. Method:	GPC
Injection #:	36	Date Processed:	2015/9/24 16:46:41 CST
Injection Volume:	20.00 ul	Channel Name:	410
Run Time:	34.00 Minutes	Sample Set Name	

Autoscaled Chromatogram

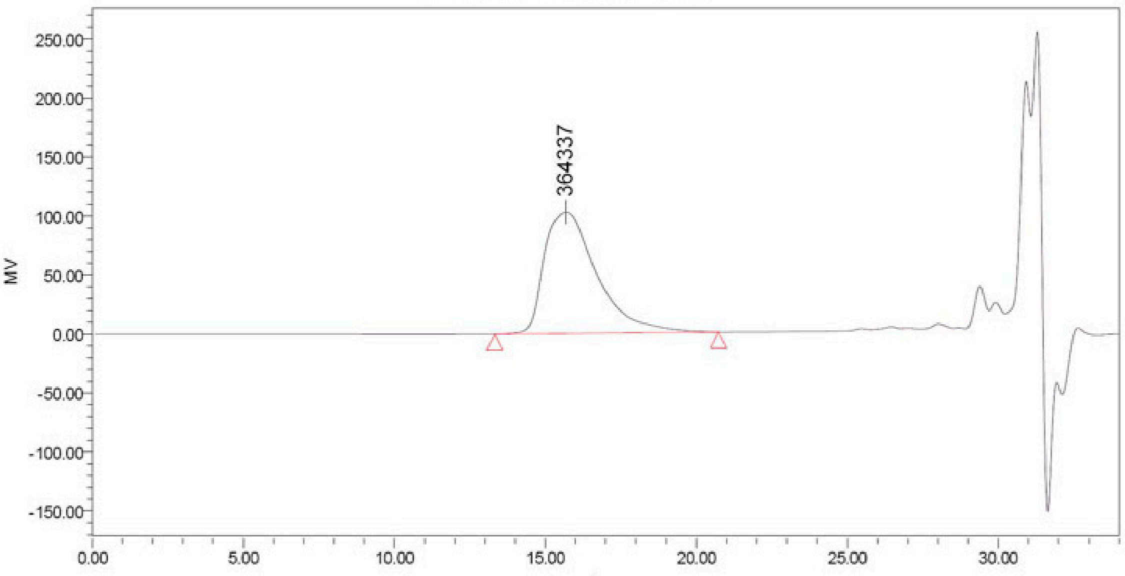

GPC Results

	Dist Name	Elution Volume (ml)	Retention Time (min)	Adjusted RT (min)	Mn	Mw	MP	Mz	Mz+1	Mz/Mw
1		15.693	15.693	15.693	182471	293691	364337	377308	435277	1.284711

GPC Results

	Mz+1/Mw	Area (*sec)	% Area	Height	% Height	Integration Type	Peak Codes	Points Across Peak	Start Time (min)	End Time (min)
1	1.482089	12432906	100.00	102733	100.00	Bb		889	13.325	20.733

Figure S20. GPC curves of Polyisoprene (Table 1, Entry 10).

Project Name 20141125
Reported by User: Breeze user (Breeze)

SAMPLE INFORMATION

Sample Name:	TS-12-5	Acquired By:	Breeze
Sample Type:		Date Acquired:	2016/4/20 2:13:38 CST
Vial:	1:B,1	Acq. Method:	
Injection #:	1	Date Processed:	2016/4/20 8:58:44 CST
Injection Volume:	20.00 ul	Channel Name:	410
Run Time:	25.00 Minutes	Channel Desc.:	
Column Type:		Sample Set Name	20160419

Broad Unknown Relative Chromatogram

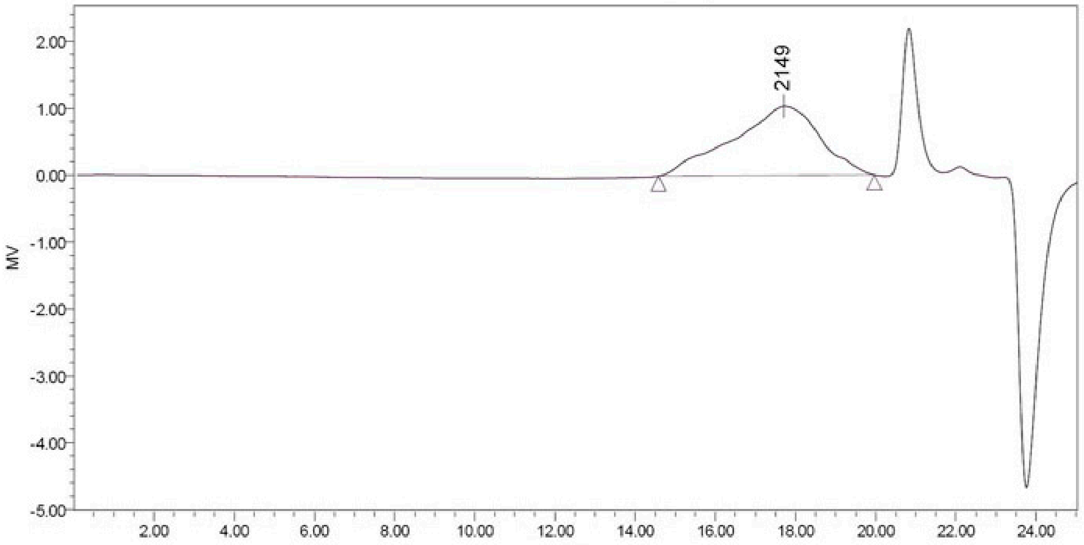

Broad Unknown Relative Peak Table

	Distribution Name	Mn (Daltons)	Mw (Daltons)	MP (Daltons)	Mz (Daltons)	Mz+1 (Daltons)	Polydispersity	Mz/Mw	Mz+1/Mw
1		1531	7287	2149	25170	41976	4.759592	3.454307	5.760811

Figure S21. GPC curves of Polyisoprene (Table 2, Entry 2).

Project Name 20141125
Reported by User: Breeze user (Breeze)

SAMPLE INFORMATION

Sample Name:	TS-14-2	Acquired By:	Breeze
Sample Type:		Date Acquired:	2016/4/20 5:36:34 CST
Vial:	1:B,8	Acq. Method:	
Injection #:	1	Date Processed:	2016/4/20 9:00:51 CST
Injection Volume:	20.00 ul	Channel Name:	410
Run Time:	25.00 Minutes	Channel Desc.:	
Column Type:		Sample Set Name	20160419

Broad Unknown Relative Chromatogram

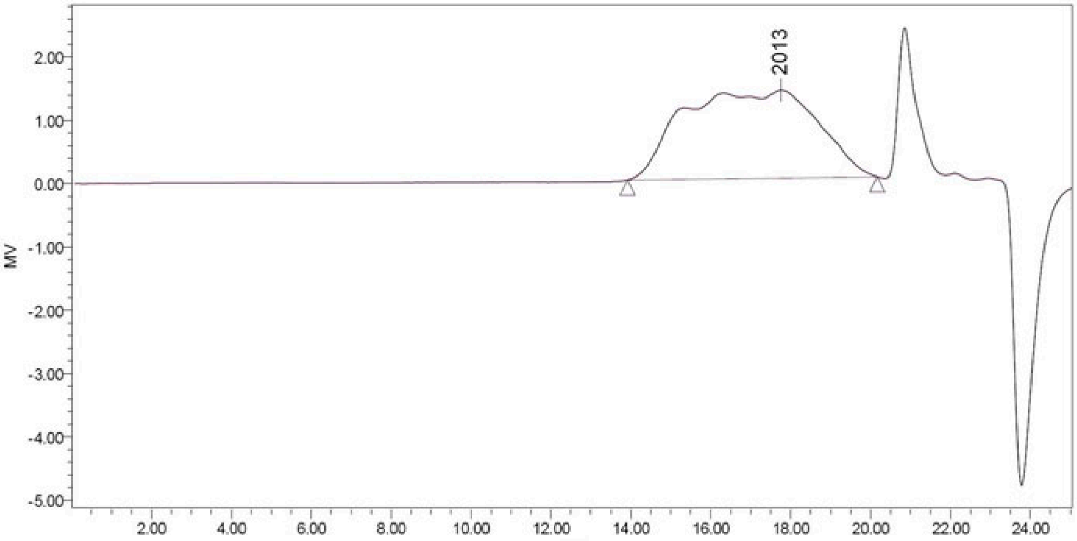

Broad Unknown Relative Peak Table

	Distribution Name	Mn (Daltons)	Mw (Daltons)	MP (Daltons)	Mz (Daltons)	Mz+1 (Daltons)	Polydispersity	Mz/Mw	Mz+1/Mw
1		1771	16620	2013	55297	86688	9.385238	3.327097	5.215833

Figure S22. GPC curves of Polyisoprene (Table 2, Entry 4).

5. NMR Spectra of the Representive Polyisoprenes

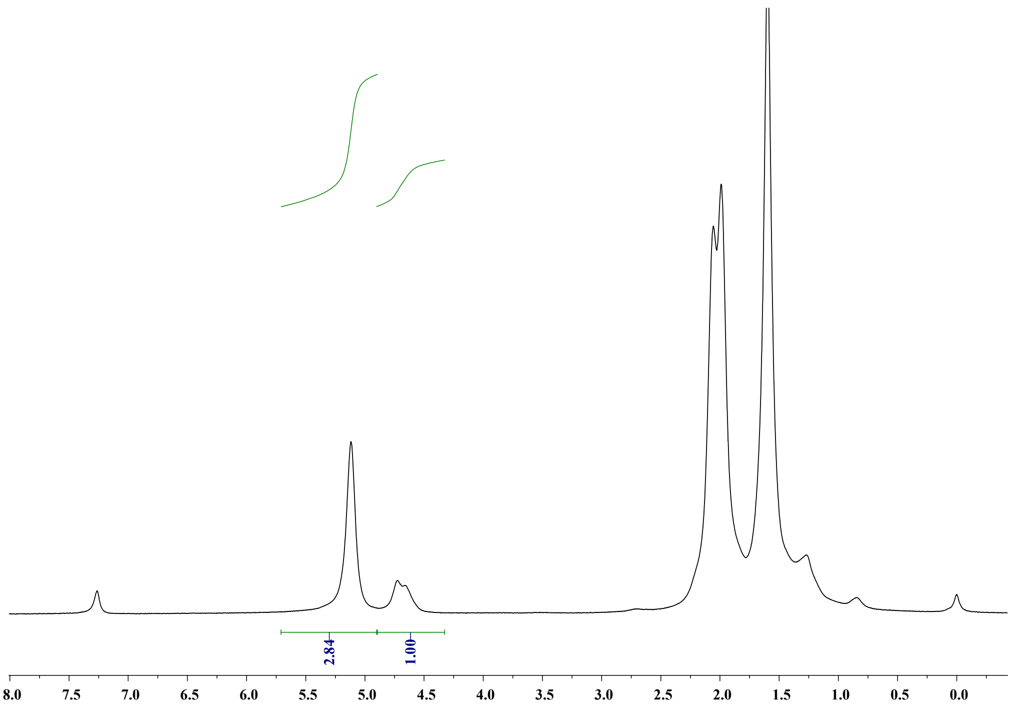

Figure S23. ^{1}H NMR spectrum (500 MHz) of polyisoprene in $CDCl_3$ (Table 1, Entry 5).

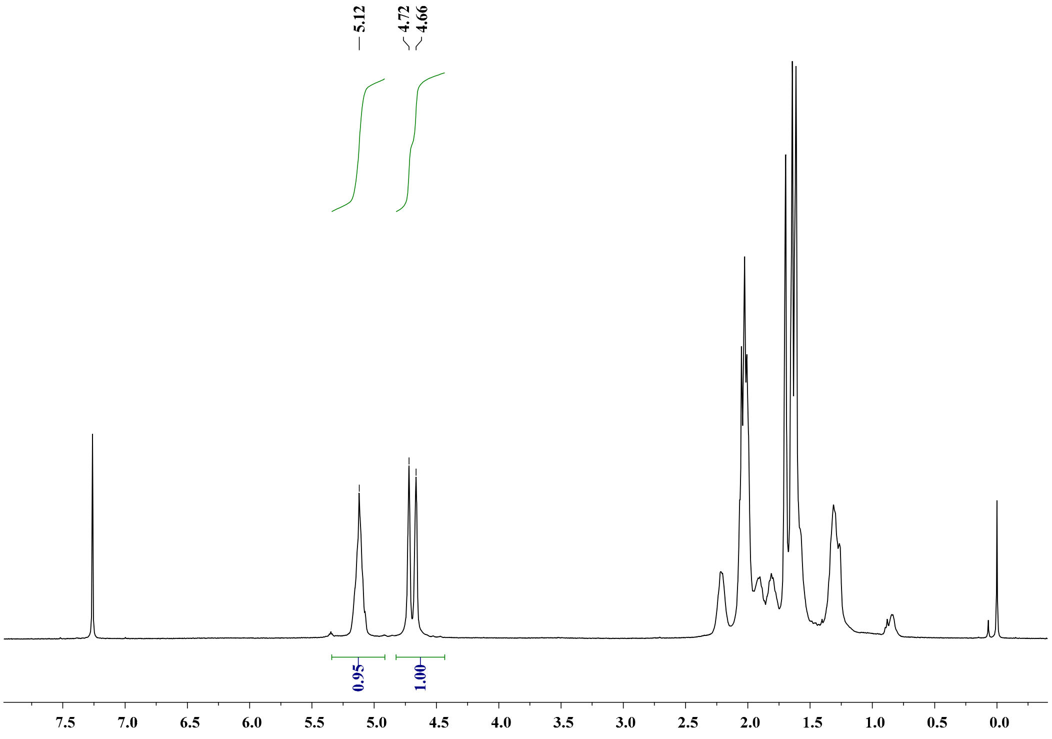

Figure S24. ^{1}H NMR spectrum (500 MHz) of polyisoprene in $CDCl_3$ (Table 1, Entry 8).

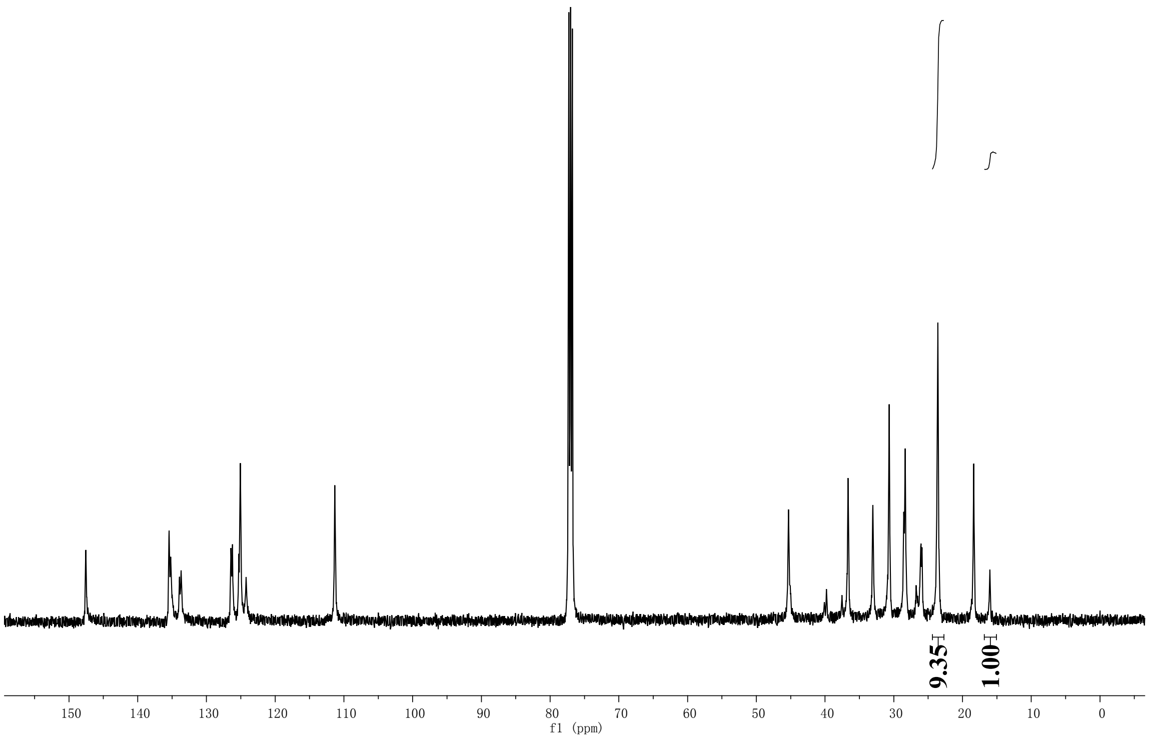

Figure S25. ^{13}C NMR spectrum (126 MHz) of polyisoprene in $CDCl_3$ (Table 1, Entry 5).

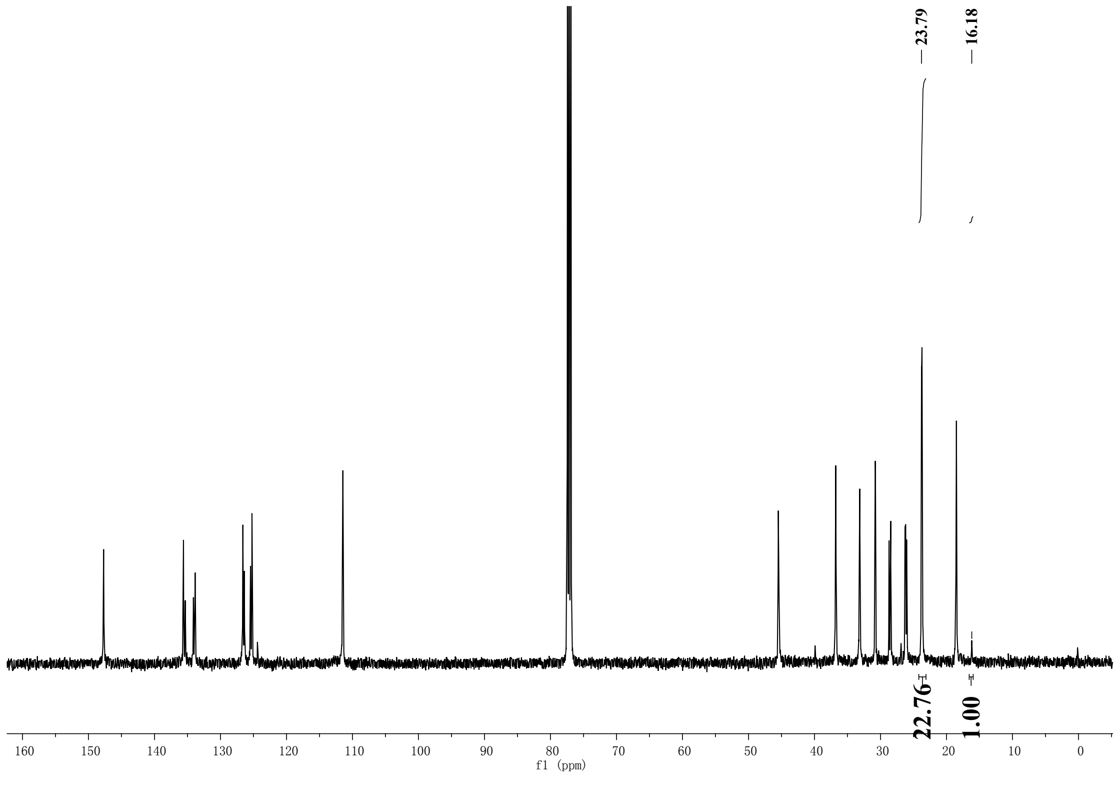

Figure S26. ^{13}C NMR spectrum (126 MHz) of polyisoprene in $CDCl_3$ (Table 1, Entry 8).

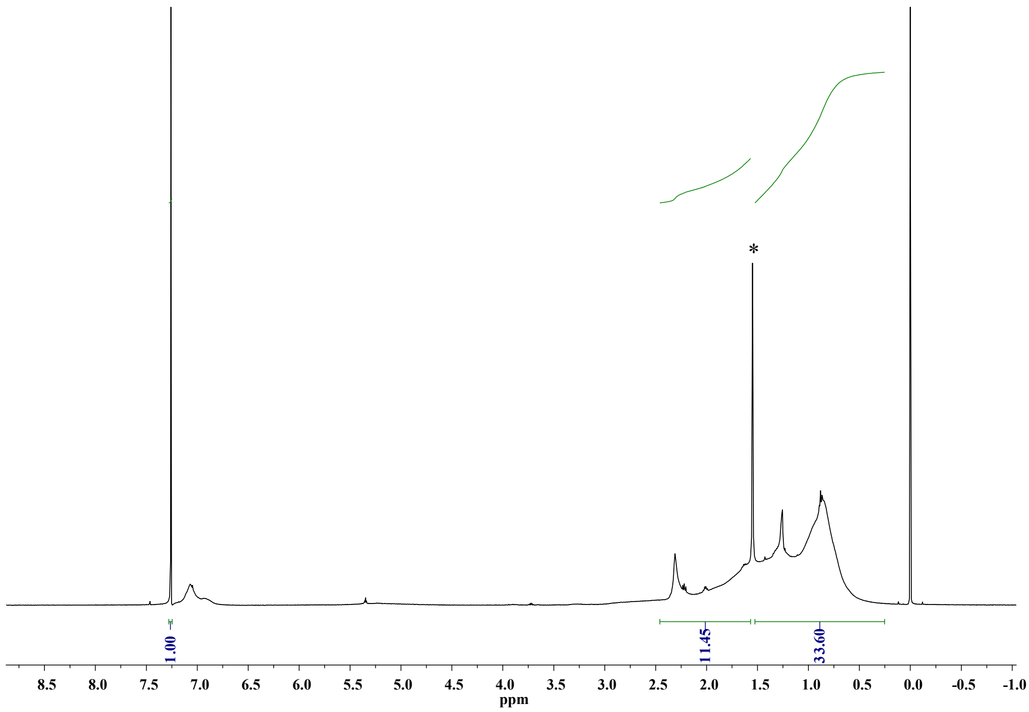

Figure S27. ^{1}H NMR spectrum (500 MHz) of polyisoprene in $CDCl_3$ (Table 2, Entry 2). * H_2O.

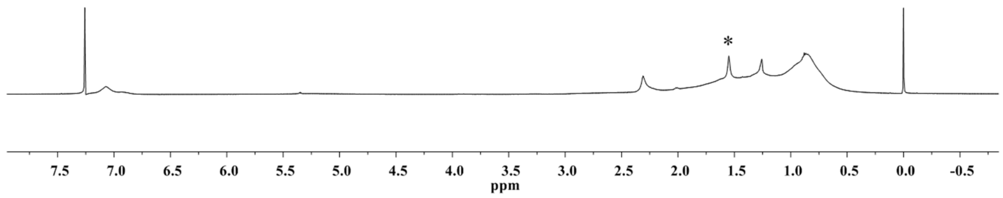

Figure S28. ^{1}H NMR spectrum (500 MHz) of polyisoprene in $CDCl_3$ (Table 2, Entry 4). * H_2O.

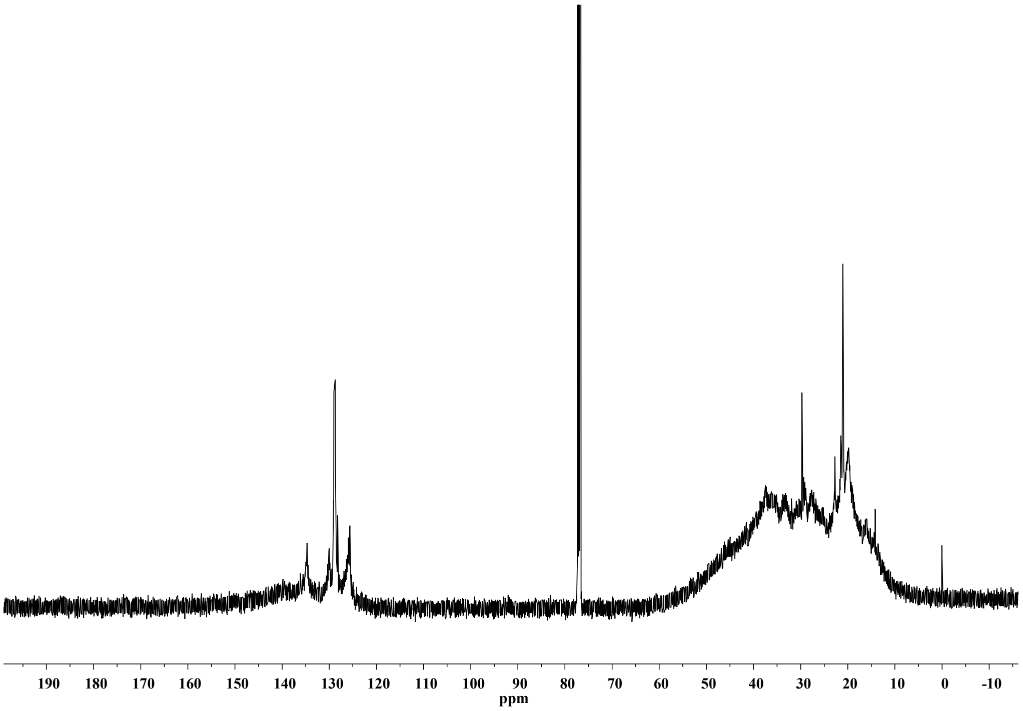

Figure S29. ^{13}C NMR spectrum (126 MHz) of polyisoprene in $CDCl_3$ (Table 2, Entry 2).

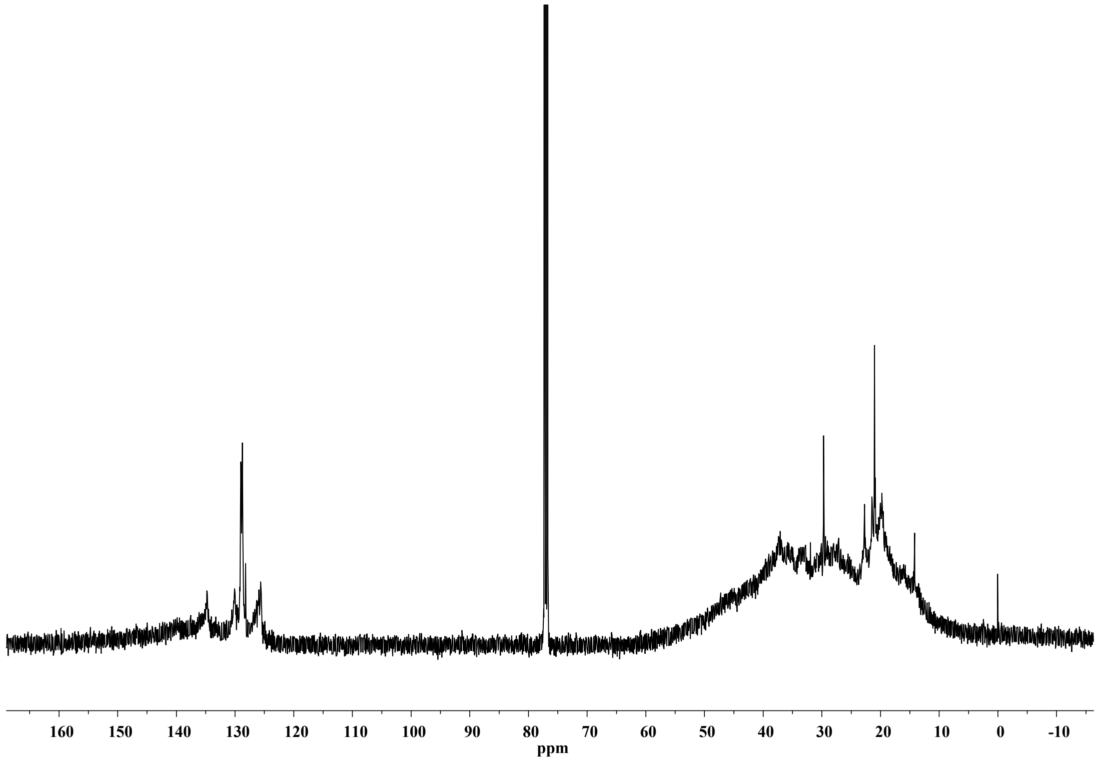

Figure S30. ^{13}C NMR spectrum (126 MHz) of polyisoprene in $CDCl_3$ (Table 2, Entry 4).

6. FTIR Spectra of Representive Polyisoprenes

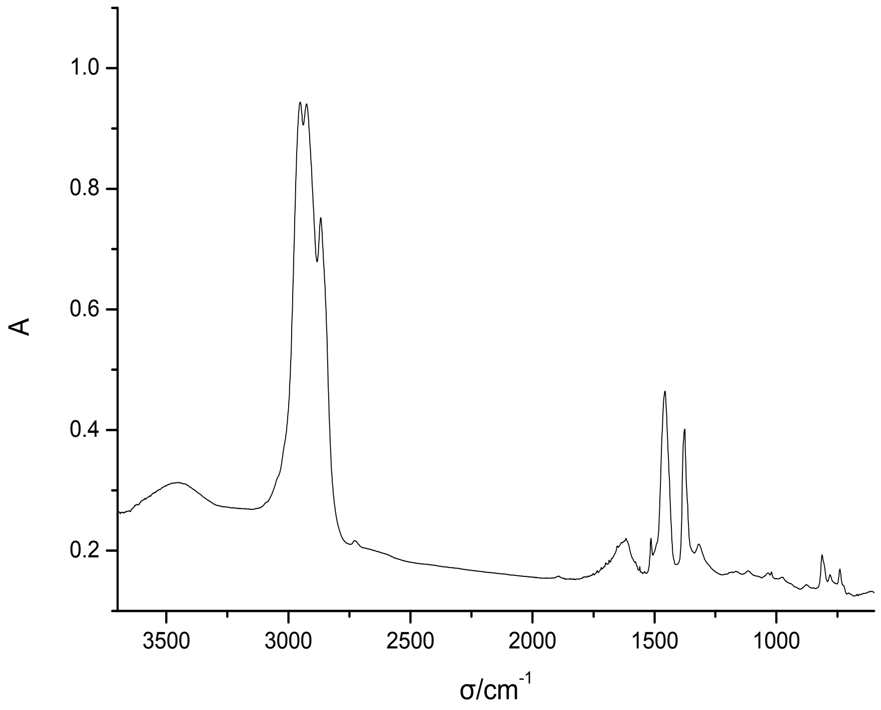

Figure S31. FTIR spectra of polyisoprene (Table 2, Entry 1).

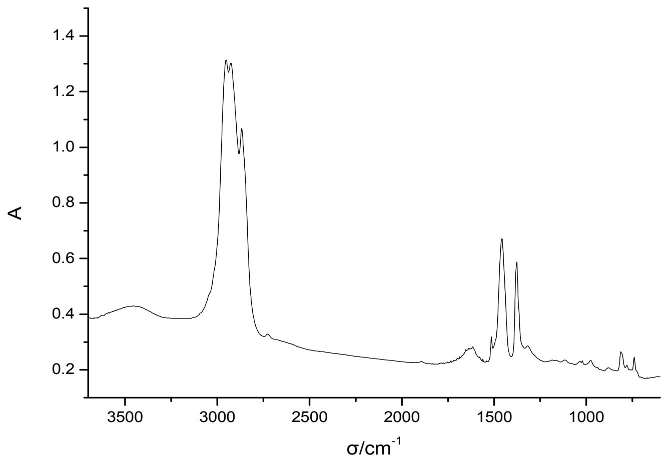

Figure S32. FTIR spectra of polyisoprene (Table 2, Entry 2).

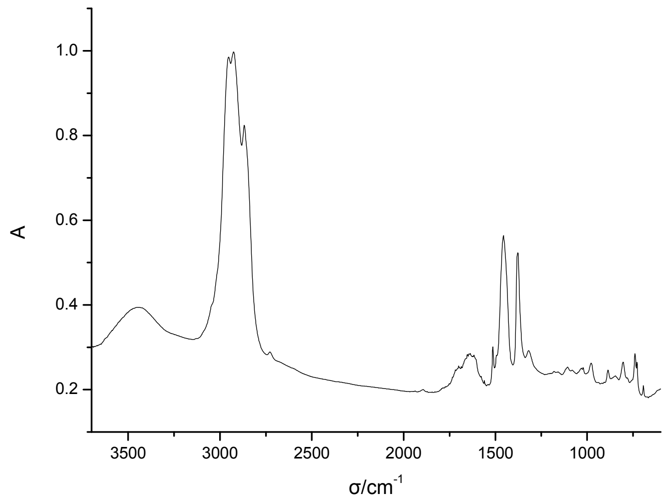

Figure S33. FTIR spectra of polyisoprene (Table 2, Entry 3).

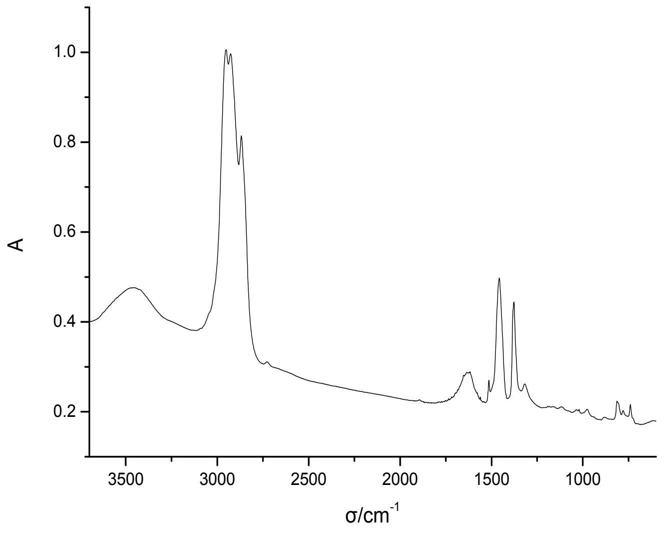

Figure S34. FTIR spectra of polyisoprene (Table 2, Entry 4).

7. X-Ray Crystallography of Complexes

CCDC numbers of **7a′** and **2b** are 1503575 and 1503576 respectively. These data can be obtained free of charge from The Cambridge Crystallographic Data Centre via www.ccdc.cam.ac.uk/ data_request/cif.

Table S2. Crystal data and structure refinement for **7a′** and **2b**.

**Identification Code**	**7a′**	**2b**
Empirical formula	$C_{78} H_{65} Cl_5 Fe_2 N_4$	$C_{14} H_{22} Cl_2 Co N_2$
Formula weight	1,347.29	348.17
Temperature/K	298(2)	298(2)
Crystal system	monoclinic	Triclinic
Space group	$P2_1/c$	P-1
a/Å	15.6440(14)	7.2174(6)
b/Å	17.5271(16)	7.9139(7)
c/Å	27.172(3)	17.2103(15)
α/°	90	78.037(2)
β/°	111.962(3)	78.580(2)
γ/°	90	65.6660(10
Volume/Å^3	6,909.7(11)	869.21(13)
Z	4	2
ρ_{calc}g/cm^3	1.295	1.330
Absorp coeff/mm^{-1}	0.659	1.284
F(000)	2,792	362
Crystal size/mm^3	0.23 × 0.18 × 0.12 mm^3	0.45 × 0.16 × 0.15
2θ range for data collection/°	2.32 to 25.02	2.85 to 25.02
Index ranges	$-18 \le h \le 17$, $-20 \le k \le 20$, $-21 \le l \le 32$	$-8 \le h \le 8$, $-9 \le k \le 9$, $-20 \le l \le 13$
Reflections collected	12,065	4,427
Independent reflections	12,066 [R(int) = 0.0000]	3,016 [R(int) = 0.0311]
Data/restraints/parameters	12,066/0/805	3,016/0/177
Goodness-of-fit on F^2	0.843	1.067
Final R indexes ($I \ge 2\sigma$ (I))	$R_1 = 0.0887$, $wR_2 = 0.2048$	$R_1 = 0.0525$, $wR_2 = 0.1142$
Final R indexes (all data)	$R_1 = 0.1808$, $wR_2 = 0.2417$	$R_1 = 0.0764$, $wR_2 = 0.1214$
Largest diff. peak/hole/e·Å^{-3}	0.47/−0.72	0.59/−0.49

Table S3. Bond Lengths (Å) and angles (°) for **7a′**.

Fe(1)–N(3)	2.156(6)
Fe(1)–N(1)	2.167(7)
Fe(1)–N(4)	2.174(6)
Fe(1)–N(2)	2.192(6)
Fe(1)–Cl(1)	2.249(2)
Fe(2)–Cl(4)	2.166(3)
Fe(2)–Cl(5)	2.181(3)
Fe(2)–Cl(3)	2.181(3)
Fe(2)–Cl(2)	2.189(3)
N(1)–C(2)	1.314(10)
N(1)–C(6)	1.331(10)
N(2)–C(1)	1.267(9)
N(2)–C(7)	1.441(9)
N(3)–C(45)	1.306(9)
N(3)–C(41)	1.366(9)

N(4)–C(40)	1.272(9)
N(4)–C(46)	1.445(9)
C(1)–C(2)	1.498(11)
C(1)–H(1)	0.9300
C(2)–C(3)	1.359(11)
C(3)–C(4)	1.365(11)
C(3)–H(3)	0.9300
C(4)–C(5)	1.391(12)
C(4)–H(4)	0.9300
C(5)–C(6)	1.372(12)
C(5)–H(5)	0.9300
C(6)–H(6)	0.9300
C(7)–C(8)	1.397(10)
C(7)–C(12)	1.404(10)
C(8)–C(9)	1.380(10)
C(8)–C(13)	1.519(11)
C(9)–C(10)	1.416(11)
C(9)–H(9)	0.9300
C(10)–C(11)	1.361(10)
C(10)–C(39)	1.507(10)
C(11)–C(12)	1.405(10)
C(11)–H(11)	0.9300
C(12)–C(26)	1.529(10)
C(13)–C(20)	1.504(11)
C(13)–C(14)	1.526(11)
C(13)–H(13)	0.9800
C(14)–C(15)	1.351(12)
C(14)–C(19)	1.407(12)
C(15)–C(16)	1.389(12)
C(15)–H(15)	0.9300
C(16)–C(17)	1.333(13)
C(16)–H(16)	0.9300
C(17)–C(18)	1.337(14)
C(17)–H(17)	0.9300
C(18)–C(19)	1.357(13)
C(18)–H(18)	0.9300
C(19)–H(19)	0.9300
C(20)–C(25)	1.372(12)
C(20)–C(21)	1.381(12)
C(21)–C(22)	1.344(14)
C(21)–H(21)	0.9300
C(22)–C(23)	1.387(16)
C(22)–H(22)	0.9300
C(23)–C(24)	1.355(15)
C(23)–H(23)	0.9300
C(24)–C(25)	1.381(14)
C(24)–H(24)	0.9300
C(25)–H(25)	0.9300
C(26)–C(27)	1.493(11)
C(26)–C(33)	1.539(11)
C(26)–H(26)	0.9800
C(27)–C(32)	1.386(12)
C(27)–C(28)	1.401(12)

C(28)–C(29)	1.365(13)
C(28)–H(28)	0.9300
C(29)–C(30)	1.354(16)
C(29)–H(29)	0.9300
C(30)–C(31)	1.342(15)
C(30)–H(30)	0.9300
C(31)–C(32)	1.412(14)
C(31)–H(31)	0.9300
C(32)–H(32)	0.9300
C(33)–C(38)	1.352(11)
C(33)–C(34)	1.370(10)
C(33)–H(33)	0.9800
C(34)–C(35)	1.386(12)
C(34)–H(34)	0.9300
C(35)–C(36)	1.331(13)
C(35)–H(35)	0.9300
C(36)–C(37)	1.367(13)
C(36)–H(36)	0.9300
C(37)–C(38)	1.389(12)
C(37)–H(37)	0.9300
C(38)–H(38)	0.9300
C(39)–H(39A)	0.9600
C(39)–H(39B)	0.9600
C(39)–H(39C)	0.9600
C(40)–C(41)	1.439(10)
C(40)–H(40)	0.9300
C(41)–C(42)	1.375(11)
C(42)–C(43)	1.354(11)
C(42)–H(42)	0.9300
C(43)–C(44)	1.382(12)
C(43)–H(43)	0.9300
C(44)–C(45)	1.367(11)
C(44)–H(44)	0.9300
C(45)–H(45)	0.9300
C(46)–C(47)	1.396(10)
C(46)–C(51)	1.410(11)
C(47)–C(48)	1.380(10)
C(47)–C(52)	1.531(11)
C(48)–C(49)	1.376(11)
C(48)–H(48)	0.9300
C(49)–C(50)	1.374(11)
C(49)–C(78)	1.495(11)
C(50)–C(51)	1.377(10)
C(50)–H(50)	0.9300
C(51)–C(65)	1.523(11)
C(52)–C(53)	1.506(11)
C(52)–C(59)	1.525(12)
C(52)–H(52)	0.9800
C(53)–C(54)	1.364(11)
C(53)–C(58)	1.413(12)
C(54)–C(55)	1.403(12)
C(54)–H(54)	0.9300
C(55)–C(56)	1.339(13)

C(55)–H(55)	0.9300
C(56)–C(57)	1.323(14)
C(56)–H(56)	0.9300
C(57)–C(58)	1.388(13)
C(57)–H(57)	0.9300
C(58)–H(58)	0.9300
C(59)–C(60)	1.353(11)
C(59)–C(64)	1.409(12)
C(60)–C(61)	1.401(13)
C(60)–H(60)	0.9300
C(61)–C(62)	1.384(15)
C(61)–H(61)	0.9300
C(62)–C(63)	1.340(14)
C(62)–H(62)	0.9300
C(63)–C(64)	1.385(13)
C(63)–H(63)	0.9300
C(64)–H(64)	0.9300
C(65)–C(72)	1.507(11)
C(65)–C(66)	1.546(11)
C(65)–H(65)	0.9800
C(66)–C(71)	1.326(11)
C(66)–C(67)	1.379(12)
C(67)–C(68)	1.390(13)
C(67)–H(67)	0.9300
C(68)–C(69)	1.350(14)
C(68)–H(68)	0.9300
C(69)–C(70)	1.387(15)
C(69)–H(69)	0.9300
C(70)–C(71)	1.396(13)
C(70)–H(70)	0.9300
C(71)–H(71)	0.9300
C(72)–C(77)	1.380(12)
C(72)–C(73)	1.398(12)
C(73)–C(74)	1.416(13)
C(73)–H(73)	0.9300
C(74)–C(75)	1.375(15)
C(74)–H(74)	0.9300
C(75)–C(76)	1.344(15)
C(75)–H(75)	0.9300
C(76)–C(77)	1.381(13)
C(76)–H(76)	0.9300
C(77)–H(77)	0.9300
C(78)–H(78A)	0.9600
C(78)–H(78B)	0.9600
C(78)–H(78C)	0.9600
N(3)–Fe(1)–N(1)	155.5(2)
N(3)–Fe(1)–N(4)	76.3(2)
N(1)–Fe(1)–N(4)	94.1(2)
N(3)–Fe(1)–N(2)	92.8(2)
N(1)–Fe(1)–N(2)	75.2(2)
N(4)–Fe(1)–N(2)	127.9(2)
N(3)–Fe(1)–Cl(1)	102.30(18)
N(1)–Fe(1)–Cl(1)	102.20(19)

N(4)–Fe(1)–Cl(1)	112.02(18)
N(2)–Fe(1)–Cl(1)	120.04(17)
Cl(4)–Fe(2)–Cl(5)	110.85(16)
Cl(4)–Fe(2)–Cl(3)	108.07(14)
Cl(5)–Fe(2)–Cl(3)	110.93(13)
Cl(4)–Fe(2)–Cl(2)	109.68(13)
Cl(5)–Fe(2)–Cl(2)	109.85(13)
Cl(3)–Fe(2)–Cl(2)	107.38(13)
C(2)–N(1)–C(6)	115.8(7)
C(2)–N(1)–Fe(1)	116.3(5)
C(6)–N(1)–Fe(1)	127.8(6)
C(1)–N(2)–C(7)	115.7(7)
C(1)–N(2)–Fe(1)	114.9(5)
C(7)–N(2)–Fe(1)	129.1(5)
C(45)–N(3)–C(41)	116.0(7)
C(45)–N(3)–Fe(1)	130.2(6)
C(41)–N(3)–Fe(1)	113.7(5)
C(40)–N(4)–C(46)	119.4(7)
C(40)–N(4)–Fe(1)	113.9(5)
C(46)–N(4)–Fe(1)	126.4(5)
N(2)–C(1)–C(2)	118.8(8)
N(2)–C(1)–H(1)	120.6
C(2)–C(1)–H(1)	120.6
N(1)–C(2)–C(3)	125.3(8)
N(1)–C(2)–C(1)	114.7(7)
C(3)–C(2)–C(1)	119.9(8)
C(2)–C(3)–C(4)	118.6(9)
C(2)–C(3)–H(3)	120.7
C(4)–C(3)–H(3)	120.7
C(3)–C(4)–C(5)	118.0(9)
C(3)–C(4)–H(4)	121.0
C(5)–C(4)–H(4)	121.0
C(6)–C(5)–C(4)	118.3(9)
C(6)–C(5)–H(5)	120.9
C(4)–C(5)–H(5)	120.9
N(1)–C(6)–C(5)	123.9(9)
N(1)–C(6)–H(6)	118.1
C(5)–C(6)–H(6)	118.1
C(8)–C(7)–C(12)	123.0(7)
C(8)–C(7)–N(2)	119.5(7)
C(12)–C(7)–N(2)	117.4(7)
C(9)–C(8)–C(7)	117.4(7)
C(9)–C(8)–C(13)	121.6(7)
C(7)–C(8)–C(13)	121.0(7)
C(8)–C(9)–C(10)	121.6(8)
C(8)–C(9)–H(9)	119.2
C(10)–C(9)–H(9)	119.2
C(11)–C(10)–C(9)	118.8(7)
C(11)–C(10)–C(39)	121.8(7)
C(9)–C(10)–C(39)	119.3(8)
C(10)–C(11)–C(12)	122.5(7)
C(10)–C(11)–H(11)	118.7
C(12)–C(11)–H(11)	118.7

C(7)–C(12)–C(11)	116.5(7)
C(7)–C(12)–C(26)	121.4(7)
C(11)–C(12)–C(26)	122.0(7)
C(20)–C(13)–C(8)	112.0(7)
C(20)–C(13)–C(14)	112.7(7)
C(8)–C(13)–C(14)	112.2(7)
C(20)–C(13)–H(13)	106.5
C(8)–C(13)–H(13)	106.5
C(14)–C(13)–H(13)	106.5
C(15)–C(14)–C(19)	117.8(9)
C(15)–C(14)–C(13)	119.9(8)
C(19)–C(14)–C(13)	122.1(9)
C(14)–C(15)–C(16)	122.5(10)
C(14)–C(15)–H(15)	118.8
C(16)–C(15)–H(15)	118.8
C(17)–C(16)–C(15)	119.1(11)
C(17)–C(16)–H(16)	120.4
C(15)–C(16)–H(16)	120.4
C(16)–C(17)–C(18)	118.7(11)
C(16)–C(17)–H(17)	120.6
C(18)–C(17)–H(17)	120.6
C(17)–C(18)–C(19)	124.8(11)
C(17)–C(18)–H(18)	117.6
C(19)–C(18)–H(18)	117.6
C(18)–C(19)–C(14)	117.0(10)
C(18)–C(19)–H(19)	121.5
C(14)–C(19)–H(19)	121.5
C(25)–C(20)–C(21)	116.1(9)
C(25)–C(20)–C(13)	124.0(9)
C(21)–C(20)–C(13)	119.9(8)
C(22)–C(21)–C(20)	123.2(11)
C(22)–C(21)–H(21)	118.4
C(20)–C(21)–H(21)	118.4
C(21)–C(22)–C(23)	119.0(12)
C(21)–C(22)–H(22)	120.5
C(23)–C(22)–H(22)	120.5
C(24)–C(23)–C(22)	119.7(12)
C(24)–C(23)–H(23)	120.2
C(22)–C(23)–H(23)	120.2
C(23)–C(24)–C(25)	119.4(12)
C(23)–C(24)–H(24)	120.3
C(25)–C(24)–H(24)	120.3
C(20)–C(25)–C(24)	122.1(10)
C(20)–C(25)–H(25)	119.0
C(24)–C(25)–H(25)	119.0
C(27)–C(26)–C(12)	113.5(6)
C(27)–C(26)–C(33)	114.1(7)
C(12)–C(26)–C(33)	111.5(7)
C(27)–C(26)–H(26)	105.6
C(12)–C(26)–H(26)	105.6
C(33)–C(26)–H(26)	105.6
C(32)–C(27)–C(28)	118.3(9)
C(32)–C(27)–C(26)	122.5(9)

C(28)–C(27)–C(26)	119.2(8)
C(29)–C(28)–C(27)	122.0(10)
C(29)–C(28)–H(28)	119.0
C(27)–C(28)–H(28)	119.0
C(30)–C(29)–C(28)	118.4(12)
C(30)–C(29)–H(29)	120.8
C(28)–C(29)–H(29)	120.8
C(31)–C(30)–C(29)	122.4(11)
C(31)–C(30)–H(30)	118.8
C(29)–C(30)–H(30)	118.8
C(30)–C(31)–C(32)	120.4(11)
C(30)–C(31)–H(31)	119.8
C(32)–C(31)–H(31)	119.8
C(27)–C(32)–C(31)	118.4(10)
C(27)–C(32)–H(32)	120.8
C(31)–C(32)–H(32)	120.8
C(38)–C(33)–C(34)	120.4(8)
C(38)–C(33)–C(26)	121.5(8)
C(34)–C(33)–C(26)	118.1(8)
C(38)–C(33)–H(33)	90.6
C(34)–C(33)–H(33)	90.6
C(26)–C(33)–H(33)	90.6
C(33)–C(34)–C(35)	118.8(9)
C(33)–C(34)–H(34)	120.6
C(35)–C(34)–H(34)	120.6
C(36)–C(35)–C(34)	122.0(9)
C(36)–C(35)–H(35)	119.0
C(34)–C(35)–H(35)	119.0
C(35)–C(36)–C(37)	118.5(9)
C(35)–C(36)–H(36)	120.7
C(37)–C(36)–H(36)	120.7
C(36)–C(37)–C(38)	121.2(10)
C(36)–C(37)–H(37)	119.4
C(38)–C(37)–H(37)	119.4
C(33)–C(38)–C(37)	119.0(9)
C(33)–C(38)–H(38)	120.5
C(37)–C(38)–H(38)	120.5
C(10)–C(39)–H(39A)	109.5
C(10)–C(39)–H(39B)	109.5
H(39A)–C(39)–H(39B)	109.5
C(10)–C(39)–H(39C)	109.5
H(39A)–C(39)–H(39C)	109.5
H(39B)–C(39)–H(39C)	109.5
N(4)–C(40)–C(41)	120.5(7)
N(4)–C(40)–H(40)	119.8
C(41)–C(40)–H(40)	119.8
N(3)–C(41)–C(42)	122.6(7)
N(3)–C(41)–C(40)	115.4(7)
C(42)–C(41)–C(40)	121.9(8)
C(43)–C(42)–C(41)	119.3(8)
C(43)–C(42)–H(42)	120.4
C(41)–C(42)–H(42)	120.4
C(42)–C(43)–C(44)	119.0(9)

C(42)–C(43)–H(43)	120.5
C(44)–C(43)–H(43)	120.5
C(45)–C(44)–C(43)	117.9(8)
C(45)–C(44)–H(44)	121.1
C(43)–C(44)–H(44)	121.1
N(3)–C(45)–C(44)	125.3(8)
N(3)–C(45)–H(45)	117.4
C(44)–C(45)–H(45)	117.4
C(47)–C(46)–C(51)	121.4(7)
C(47)–C(46)–N(4)	118.8(7)
C(51)–C(46)–N(4)	119.8(7)
C(48)–C(47)–C(46)	117.2(7)
C(48)–C(47)–C(52)	122.3(8)
C(46)–C(47)–C(52)	120.4(7)
C(49)–C(48)–C(47)	123.1(8)
C(49)–C(48)–H(48)	118.5
C(47)–C(48)–H(48)	118.5
C(50)–C(49)–C(48)	117.9(8)
C(50)–C(49)–C(78)	121.6(8)
C(48)–C(49)–C(78)	120.5(9)
C(49)–C(50)–C(51)	122.7(8)
C(49)–C(50)–H(50)	118.6
C(51)–C(50)–H(50)	118.6
C(50)–C(51)–C(46)	117.4(8)
C(50)–C(51)–C(65)	124.1(7)
C(46)–C(51)–C(65)	118.4(7)
C(53)–C(52)–C(59)	114.2(7)
C(53)–C(52)–C(47)	114.7(7)
C(59)–C(52)–C(47)	110.1(7)
C(53)–C(52)–H(52)	105.6
C(59)–C(52)–H(52)	105.6
C(47)–C(52)–H(52)	105.6
C(54)–C(53)–C(58)	117.0(8)
C(54)–C(53)–C(52)	123.7(8)
C(58)–C(53)–C(52)	119.2(8)
C(53)–C(54)–C(55)	121.4(9)
C(53)–C(54)–H(54)	119.3
C(55)–C(54)–H(54)	119.3
C(56)–C(55)–C(54)	119.1(10)
C(56)–C(55)–H(55)	120.4
C(54)–C(55)–H(55)	120.4
C(57)–C(56)–C(55)	122.1(11)
C(57)–C(56)–H(56)	119.0
C(55)–C(56)–H(56)	119.0
C(56)–C(57)–C(58)	120.4(11)
C(56)–C(57)–H(57)	119.8
C(58)–C(57)–H(57)	119.8
C(57)–C(58)–C(53)	119.9(10)
C(57)–C(58)–H(58)	120.0
C(53)–C(58)–H(58)	120.0
C(60)–C(59)–C(64)	118.9(9)
C(60)–C(59)–C(52)	120.7(8)
C(64)–C(59)–C(52)	120.2(8)

C(59)–C(60)–C(61)	119.8(10)
C(59)–C(60)–H(60)	120.1
C(61)–C(60)–H(60)	120.1
C(62)–C(61)–C(60)	121.1(11)
C(62)–C(61)–H(61)	119.4
C(60)–C(61)–H(61)	119.4
C(63)–C(62)–C(61)	118.8(11)
C(63)–C(62)–H(62)	120.6
C(61)–C(62)–H(62)	120.6
C(62)–C(63)–C(64)	121.6(11)
C(62)–C(63)–H(63)	119.2
C(64)–C(63)–H(63)	119.2
C(63)–C(64)–C(59)	119.8(10)
C(63)–C(64)–H(64)	120.1
C(59)–C(64)–H(64)	120.1
C(72)–C(65)–C(51)	112.6(7)
C(72)–C(65)–C(66)	112.2(7)
C(51)–C(65)–C(66)	112.4(7)
C(72)–C(65)–H(65)	106.4
C(51)–C(65)–H(65)	106.4
C(66)–C(65)–H(65)	106.4
C(71)–C(66)–C(67)	120.1(9)
C(71)–C(66)–C(65)	119.2(8)
C(67)–C(66)–C(65)	120.7(8)
C(66)–C(67)–C(68)	119.7(10)
C(66)–C(67)–H(67)	120.1
C(68)–C(67)–H(67)	120.1
C(69)–C(68)–C(67)	119.4(10)
C(69)–C(68)–H(68)	120.3
C(67)–C(68)–H(68)	120.3
C(68)–C(69)–C(70)	121.3(11)
C(68)–C(69)–H(69)	119.3
C(70)–C(69)–H(69)	119.3
C(69)–C(70)–C(71)	117.3(11)
C(69)–C(70)–H(70)	121.3
C(71)–C(70)–H(70)	121.3
C(66)–C(71)–C(70)	122.0(10)
C(66)–C(71)–H(71)	119.0
C(70)–C(71)–H(71)	119.0
C(77)–C(72)–C(73)	119.1(8)
C(77)–C(72)–C(65)	119.4(8)
C(73)–C(72)–C(65)	121.5(8)
C(72)–C(73)–C(74)	119.8(10)
C(72)–C(73)–H(73)	120.1
C(74)–C(73)–H(73)	120.1
C(75)–C(74)–C(73)	118.7(10)
C(75)–C(74)–H(74)	120.6
C(73)–C(74)–H(74)	120.6
C(76)–C(75)–C(74)	120.9(10)
C(76)–C(75)–H(75)	119.5
C(74)–C(75)–H(75)	119.5
C(75)–C(76)–C(77)	121.5(11)
C(75)–C(76)–H(76)	119.3

C(77)–C(76)–H(76)	119.3
C(72)–C(77)–C(76)	119.8(10)
C(72)–C(77)–H(77)	120.1
C(76)–C(77)–H(77)	120.1
C(49)–C(78)–H(78A)	109.5
C(49)–C(78)–H(78B)	109.5
H(78A)–C(78)–H(78B)	109.5
C(49)–C(78)–H(78C)	109.5
H(78A)–C(78)–H(78C)	109.5
H(78B)–C(78)–H(78C)	109.5

Table S4. Bond Lengths (Å) and angles (°) for **2b**.

Co(1)–N(1)	2.040(3)
Co(1)–N(2)	2.046(3)
Co(1)–Cl(2)	2.2164(12)
Co(1)–Cl(1)	2.2183(13)
N(1)–C(6)	1.331(5)
N(1)–C(2)	1.345(5)
N(2)–C(1)	1.270(5)
N(2)–C(7)	1.502(5)
C(1)–C(2)	1.479(6)
C(1)–H(1)	0.9300
C(2)–C(3)	1.377(6)
C(3)–C(4)	1.380(6)
C(3)–H(3)	0.9300
C(4)–C(5)	1.350(6)
C(4)–H(4)	0.9300
C(5)–C(6)	1.387(6)
C(5)–H(5)	0.9300
C(6)–H(6)	0.9300
C(7)–C(13)	1.511(6)
C(7)–C(14)	1.520(6)
C(7)–C(8)	1.542(6)
C(8)–C(9)	1.548(6)
C(8)–H(8A)	0.9700
C(8)–H(8B)	0.9700
C(9)–C(10)	1.510(7)
C(9)–C(12)	1.535(8)
C(9)–C(11)	1.547(8)
C(10)–H(10A)	0.9600
C(10)–H(10B)	0.9600
C(10)–H(10C)	0.9600
C(11)–H(11A)	0.9600
C(11)–H(11B)	0.9600
C(11)–H(11C)	0.9600
C(12)–H(12A)	0.9600
C(12)–H(12B)	0.9600
C(12)–H(12C)	0.9600
C(13)–H(13A)	0.9600
C(13)–H(13B)	0.9600
C(13)–H(13C)	0.9600
C(14)–H(14A)	0.9600

C(14)–H(14B)	0.9600
C(14)–H(14C)	0.9600
N(1)–Co(1)–N(2)	81.61(13)
N(1)–Co(1)–Cl(2)	110.74(10)
N(2)–Co(1)–Cl(2)	117.71(9)
N(1)–Co(1)–Cl(1)	111.40(10)
N(2)–Co(1)–Cl(1)	119.08(10)
Cl(2)–Co(1)–Cl(1)	112.06(5)
C(6)–N(1)–C(2)	118.1(4)
C(6)–N(1)–Co(1)	129.8(3)
C(2)–N(1)–Co(1)	112.1(3)
C(1)–N(2)–C(7)	121.7(4)
C(1)–N(2)–Co(1)	112.0(3)
C(7)–N(2)–Co(1)	126.2(3)
N(2)–C(1)–C(2)	119.6(4)
N(2)–C(1)–H(1)	120.2
C(2)–C(1)–H(1)	120.2
N(1)–C(2)–C(3)	122.2(4)
N(1)–C(2)–C(1)	114.6(4)
C(3)–C(2)–C(1)	123.2(4)
C(2)–C(3)–C(4)	118.7(4)
C(2)–C(3)–H(3)	120.7
C(4)–C(3)–H(3)	120.7
C(5)–C(4)–C(3)	119.6(4)
C(5)–C(4)–H(4)	120.2
C(3)–C(4)–H(4)	120.2
C(4)–C(5)–C(6)	119.0(4)
C(4)–C(5)–H(5)	120.5
C(6)–C(5)–H(5)	120.5
N(1)–C(6)–C(5)	122.4(4)
N(1)–C(6)–H(6)	118.8
C(5)–C(6)–H(6)	118.8
N(2)–C(7)–C(13)	113.7(4)
N(2)–C(7)–C(14)	104.5(3)
C(13)–C(7)–C(14)	109.4(4)
N(2)–C(7)–C(8)	107.2(3)
C(13)–C(7)–C(8)	113.8(4)
C(14)–C(7)–C(8)	107.6(4)
C(7)–C(8)–C(9)	123.1(4)
C(7)–C(8)–H(8A)	106.5
C(9)–C(8)–H(8A)	106.5
C(7)–C(8)–H(8B)	106.5
C(9)–C(8)–H(8B)	106.5
H(8A)–C(8)–H(8B)	106.5
C(10)–C(9)–C(12)	112.2(5)
C(10)–C(9)–C(11)	107.9(4)
C(12)–C(9)–C(11)	107.0(5)
C(10)–C(9)–C(8)	113.9(4)
C(12)–C(9)–C(8)	110.9(4)
C(11)–C(9)–C(8)	104.3(5)
C(9)–C(10)–H(10A)	109.5
C(9)–C(10)–H(10B)	109.5
H(10A)–C(10)–H(10B)	109.5

C(9)–C(10)–H(10C)	109.5
H(10A)–C(10)–H(10C)	109.5
H(10B)–C(10)–H(10C)	109.5
C(9)–C(11)–H(11A)	109.5
C(9)–C(11)–H(11B)	109.5
H(11A)–C(11)–H(11B)	109.5
C(9)–C(11)–H(11C)	109.5
H(11A)–C(11)–H(11C)	109.5
H(11B)–C(11)–H(11C)	109.5
C(9)–C(12)–H(12A)	109.5
C(9)–C(12)–H(12B)	109.5
H(12A)–C(12)–H(12B)	109.5
C(9)–C(12)–H(12C)	109.5
H(12A)–C(12)–H(12C)	109.5
H(12B)–C(12)–H(12C)	109.5
C(7)–C(13)–H(13A)	109.5
C(7)–C(13)–H(13B)	109.5
H(13A)–C(13)–H(13B)	109.5
C(7)–C(13)–H(13C)	109.5
H(13A)–C(13)–H(13C)	109.5
H(13B)–C(13)–H(13C)	109.5
C(7)–C(14)–H(14A)	109.5
C(7)–C(14)–H(14B)	109.5
H(14A)–C(14)–H(14B)	109.5
C(7)–C(14)–H(14C)	109.5
H(14A)–C(14)–H(14C)	109.5
H(14B)–C(14)–H(14C)	109.5
